# Supplementary figures and images for: Transcriptional regulation of genes bearing intronic heterochromatin in the rice genome
Source: PLoS Genet. 2020 Mar 18;16(3):e1008637. doi: 10.1371/journal.pgen.1008637 (PMC7145194; doi:10.1371/journal.pgen.1008637)

A TAIR10 Gene models (n= 28,496)

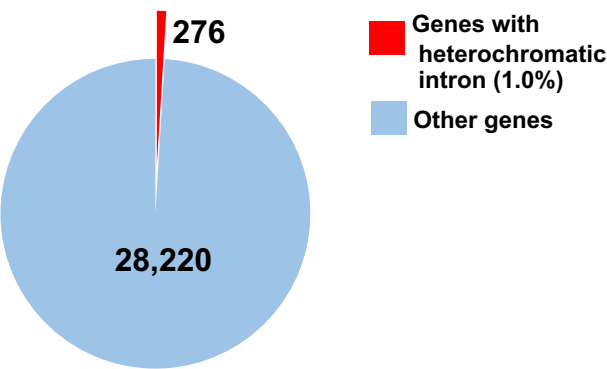

B

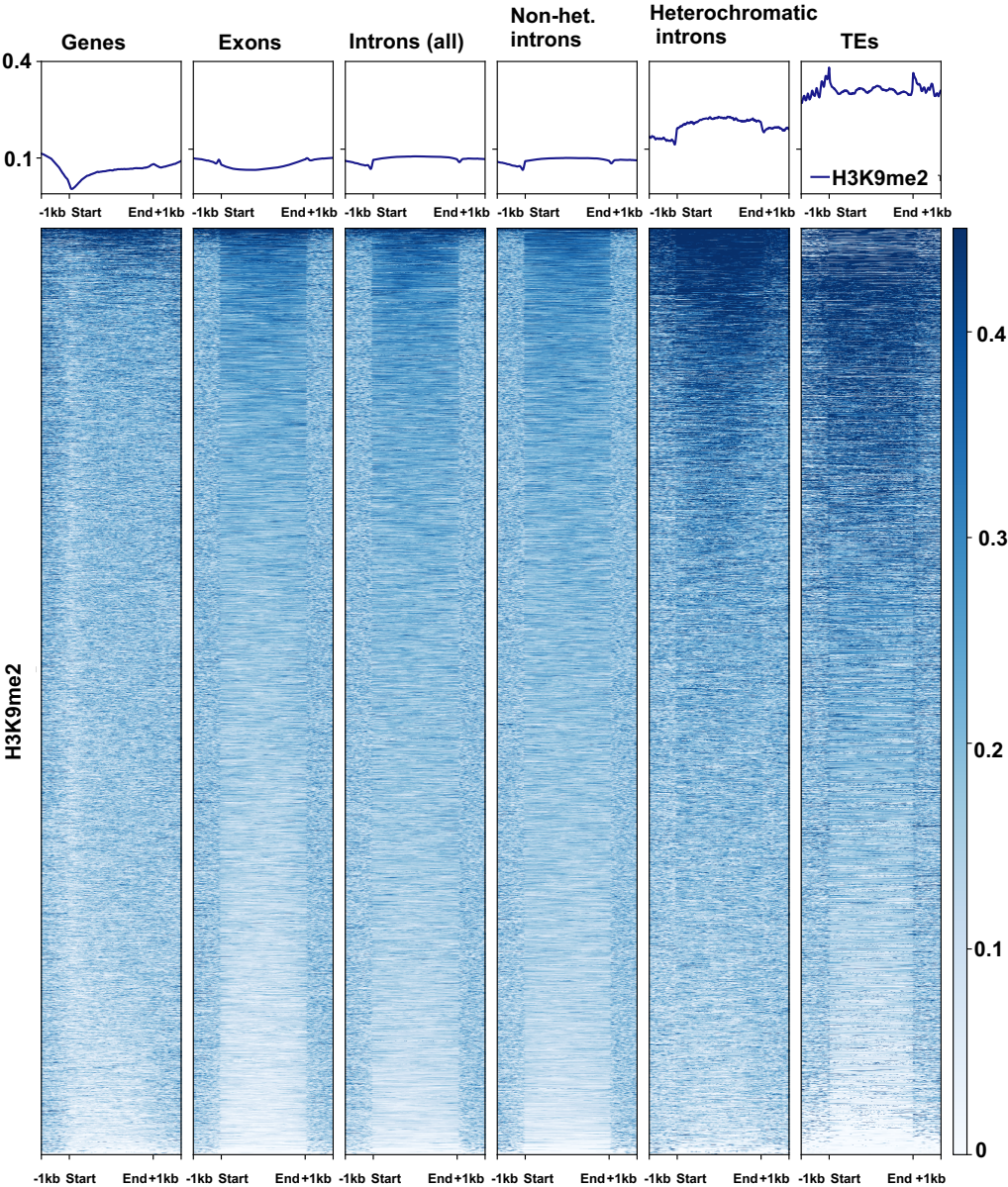

Supplement: S1 Fig — (A) Arabidopsis thaliana genes (TAIR10) containing intron with heterochromatic domains. (B) Heatmap showing accumulation of H3K9 di-methylation on genome features in the rice genome. Data from [109] were used for the analysis. (PDF) [file pgen.1008637.s001.pdf]

A

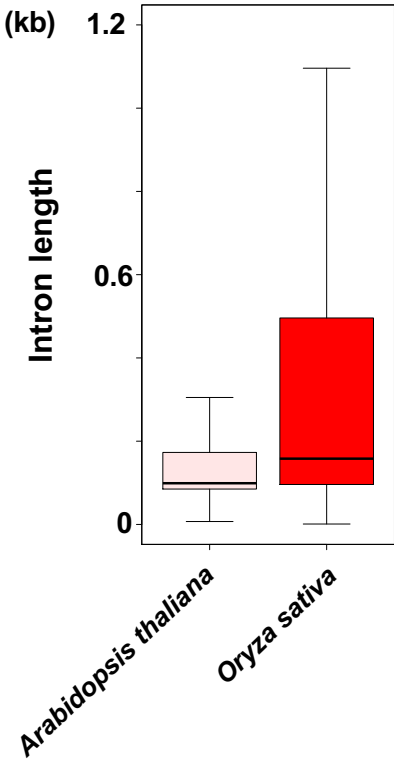

B

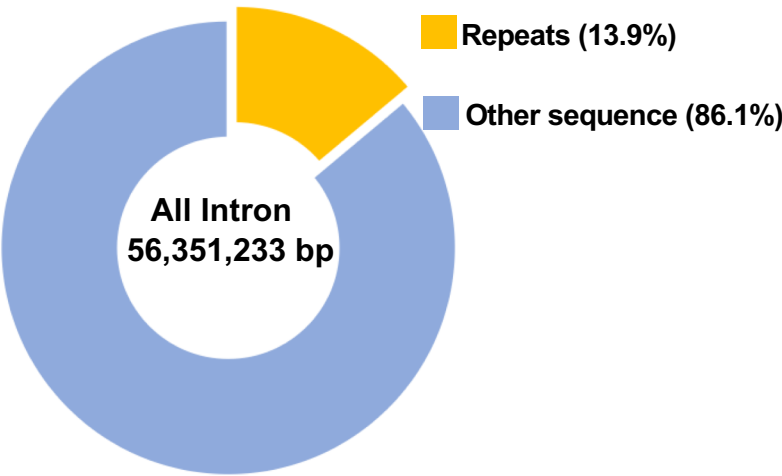

C

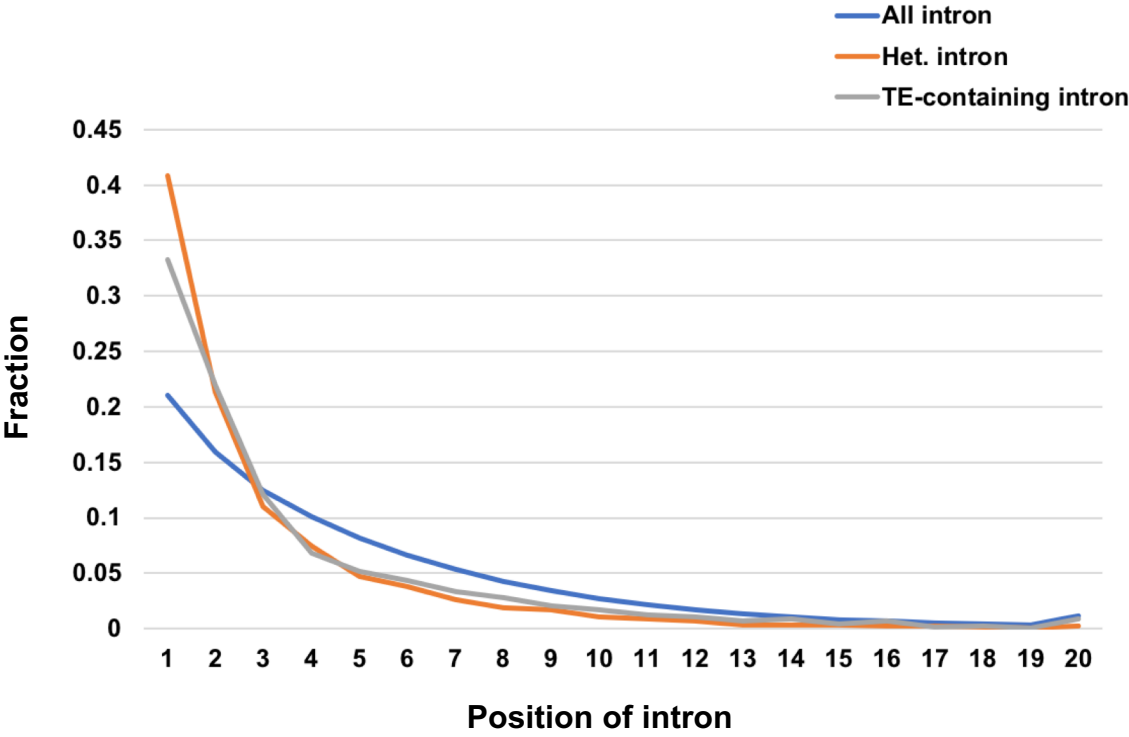

Supplement: S2 Fig — (A) A comparison of intron length between Arabidopsis thaliana (n = 127,836; average 169.0 bp) and Oryza sativa (n = 126,068; average 446.9 bp). (B) Fraction of repetitive elements in intronic regions of the rice genome. (C) Enrichment of heterochromatin and TEs in promoter-proximal introns. Fractions of all intron (n = 151,045), and heterochromatic introns (n = 6,086), and TE-containing introns (n = 1,982) are shown in the relative positions. Identical intronic regions annotated in different positions in different splicing variants were independently counted. (PDF) [file pgen.1008637.s002.pdf]

A

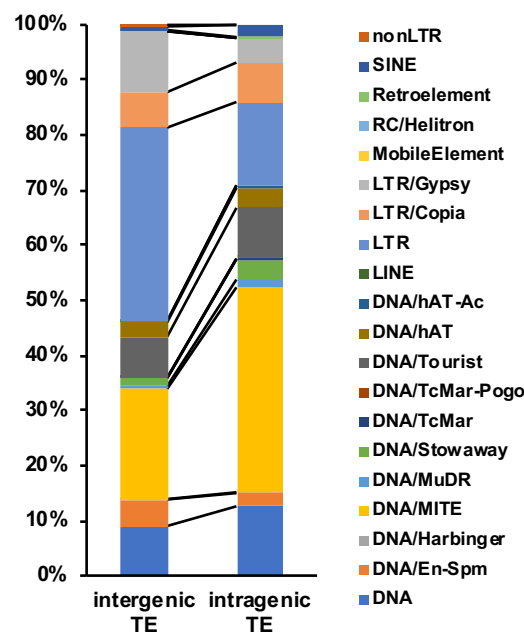

B

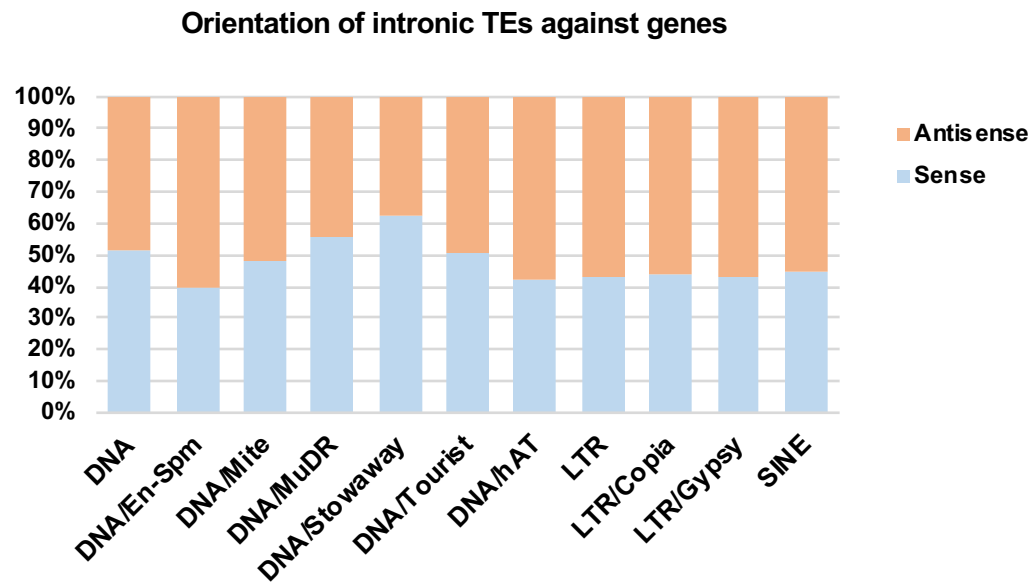

C

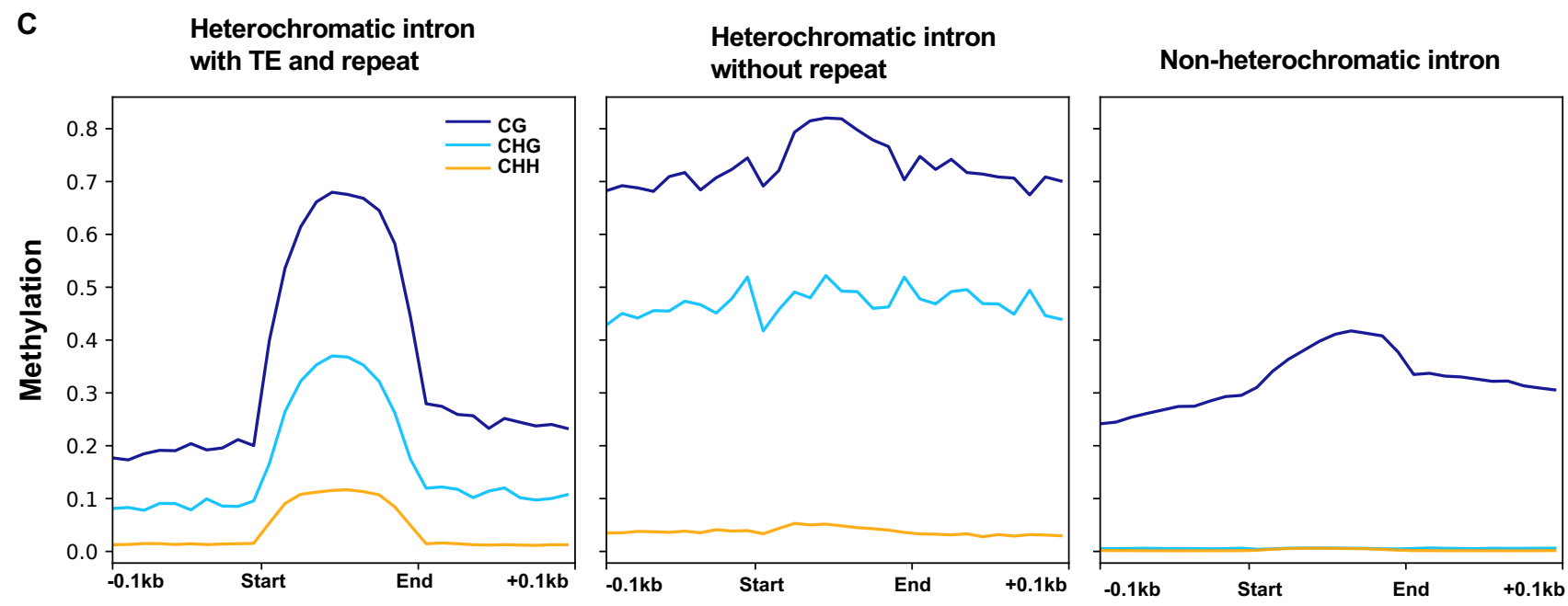

D

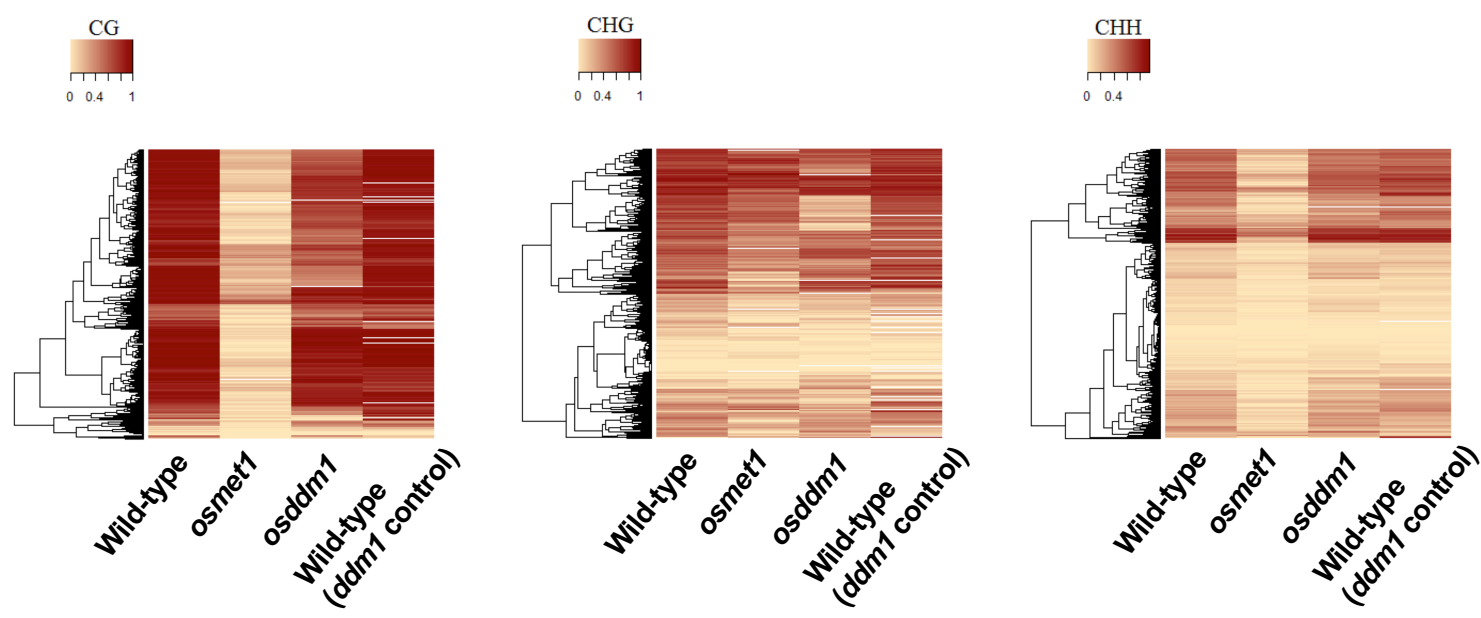

Supplement: S3 Fig — (A) Fraction of TE families in the intronic regions of the Oryza sativa genome. (B) Orientation of intronic TE insertion against gene annotations in each TE family. No significant orientation bias was observed in the TE families (p > 0.01; two-sided binominal test). (C) Metaplots of DNA methylation in CG, CHG and CHH contexts for heterochromatic introns with TEs and repeats (n = 4,886), heterochromatic introns without repeat (n = 923), and non-heterochromatic introns (n = 145,235). (D) Heatmap of methylation profiles of intronic TEs in wild-type O. sativa and mutants of OsMET1 (met1) and of OsDDM1 (ddm1) at CG, CHG, and CHH-contexts. (PDF) [file pgen.1008637.s003.pdf]

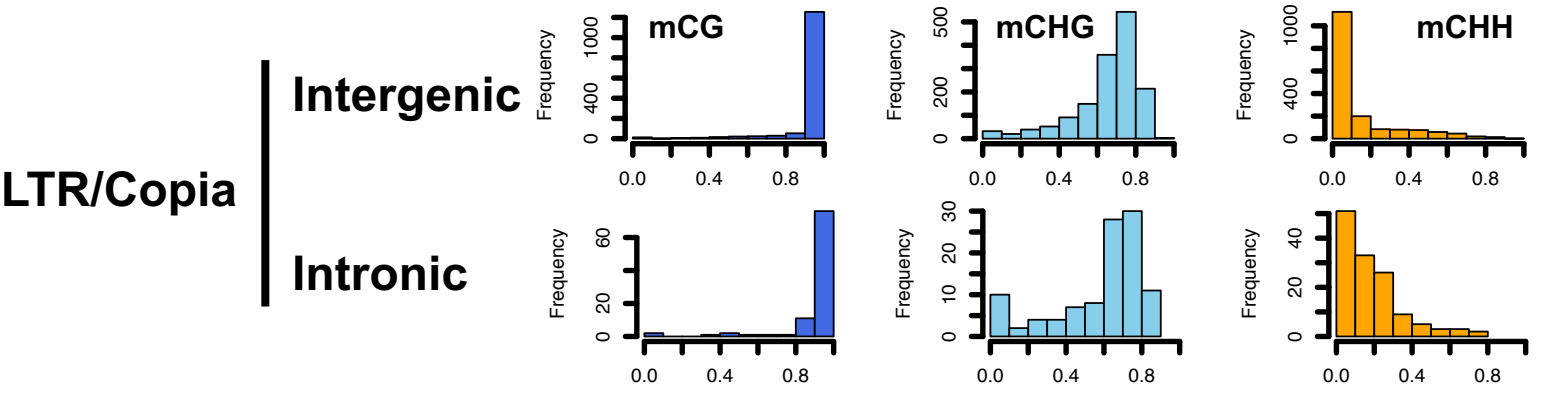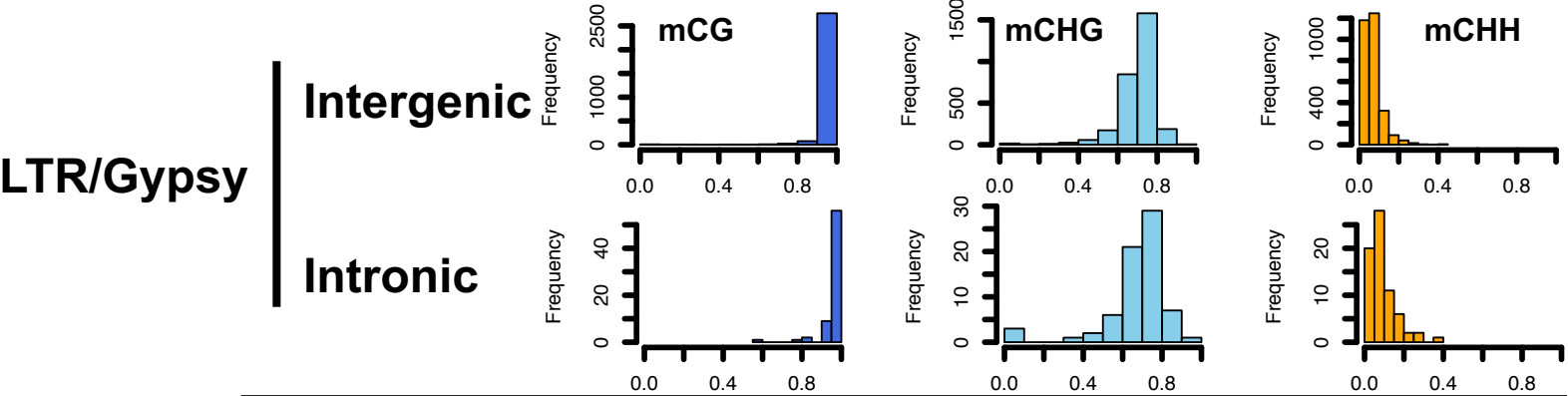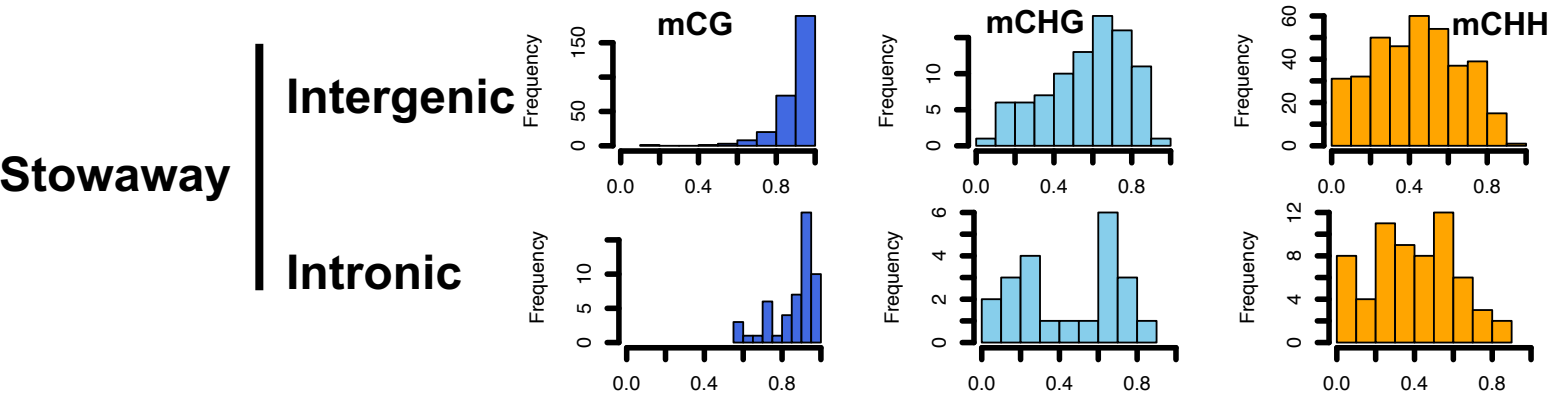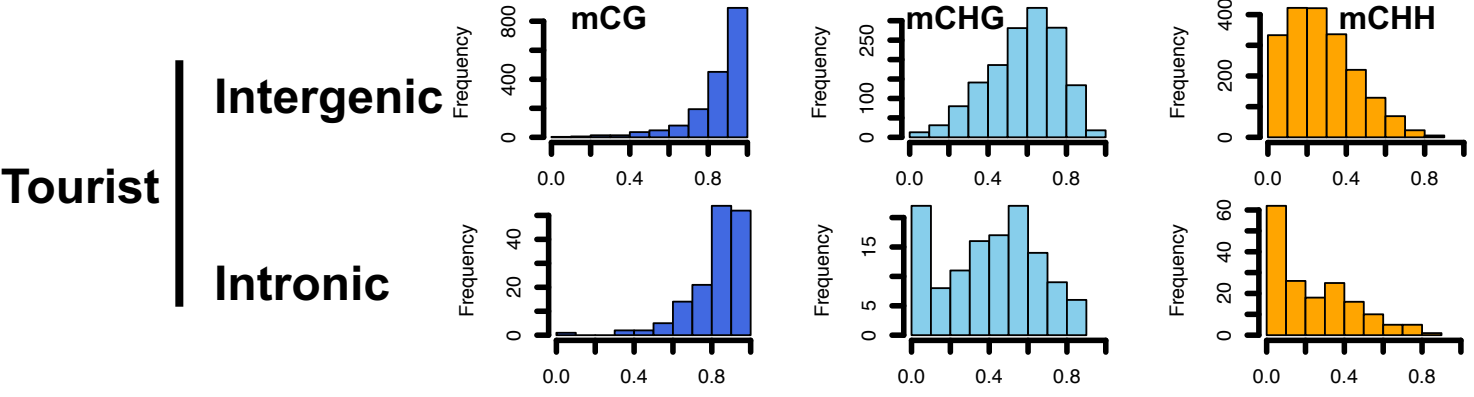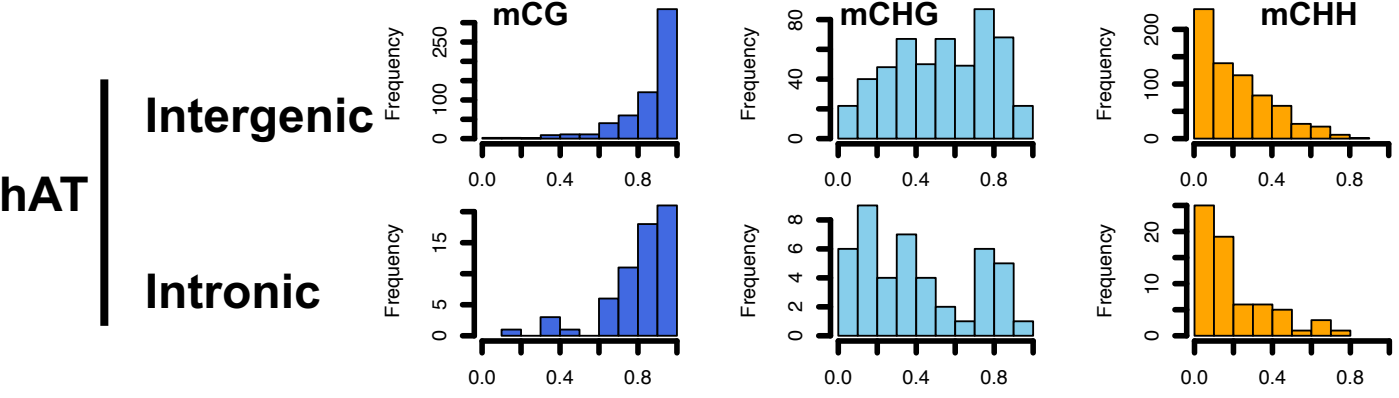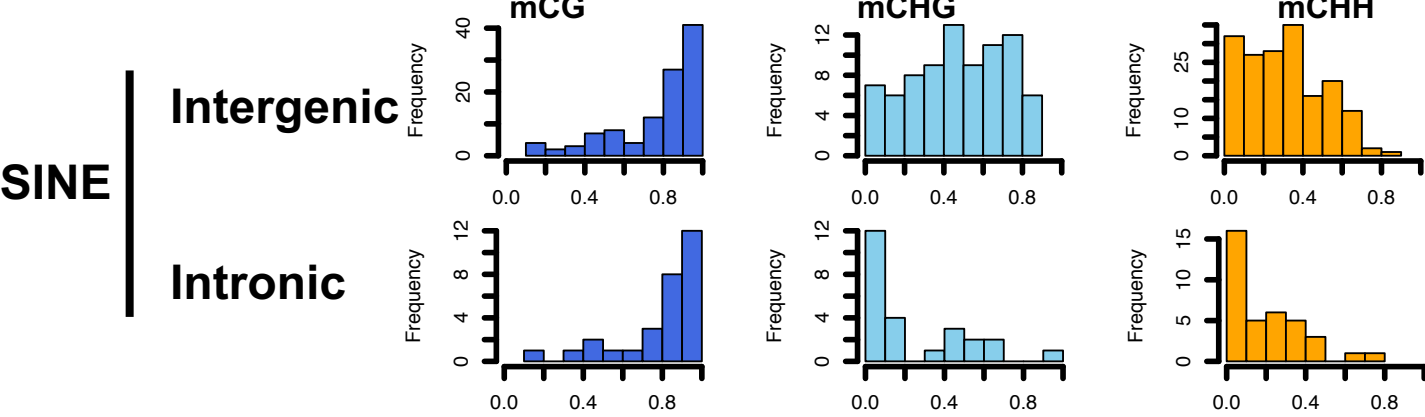

Supplement: S4 Fig — Histograms of the number of representative intergenic and intronic TE families (>20 copies in each category) and their methylation levels (0 to 1) in CG, CHG, and CHH contexts. TEs with methylation data at ≥ 5 Cs were analyzed. (PDF) [file pgen.1008637.s004.pdf]

mCG

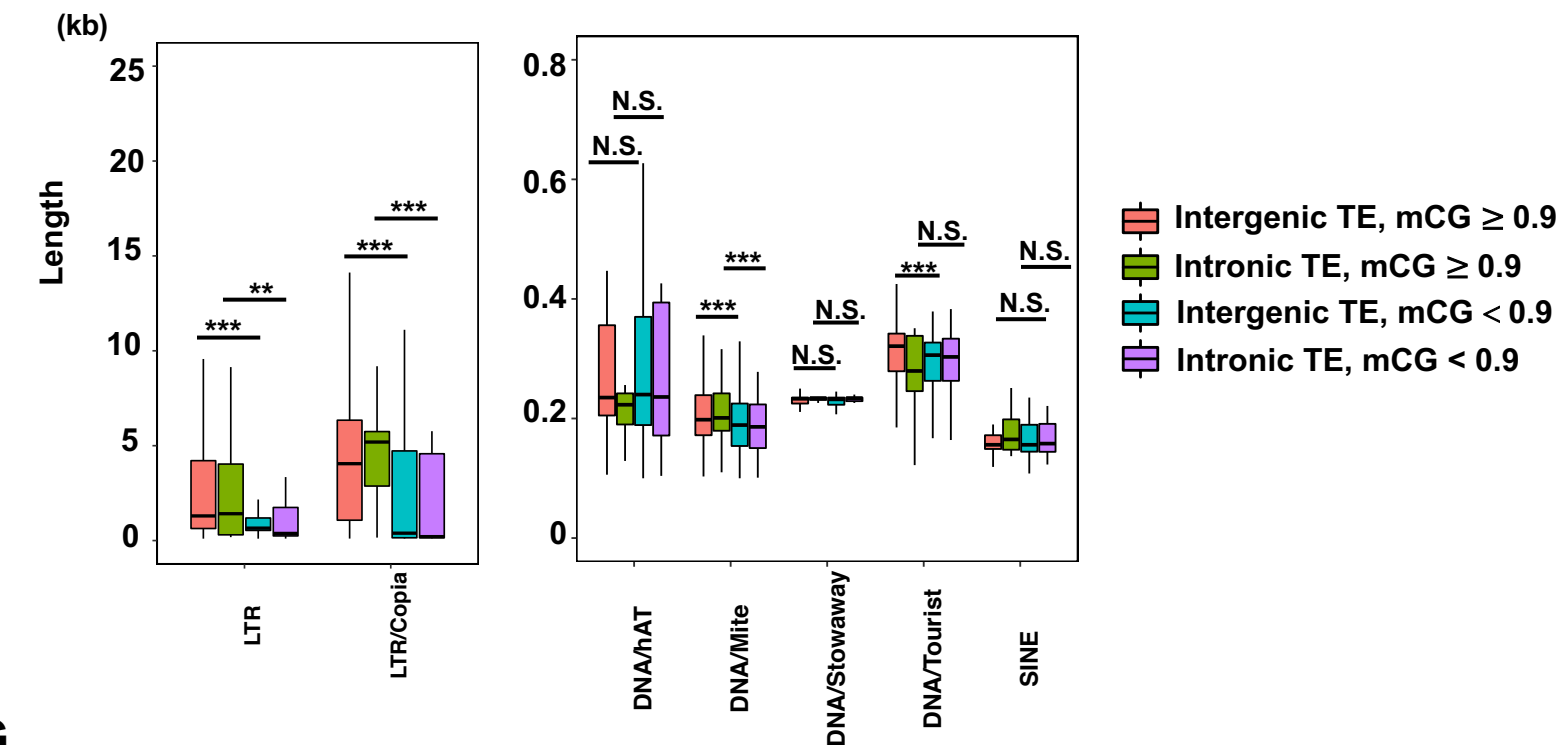

mCHG

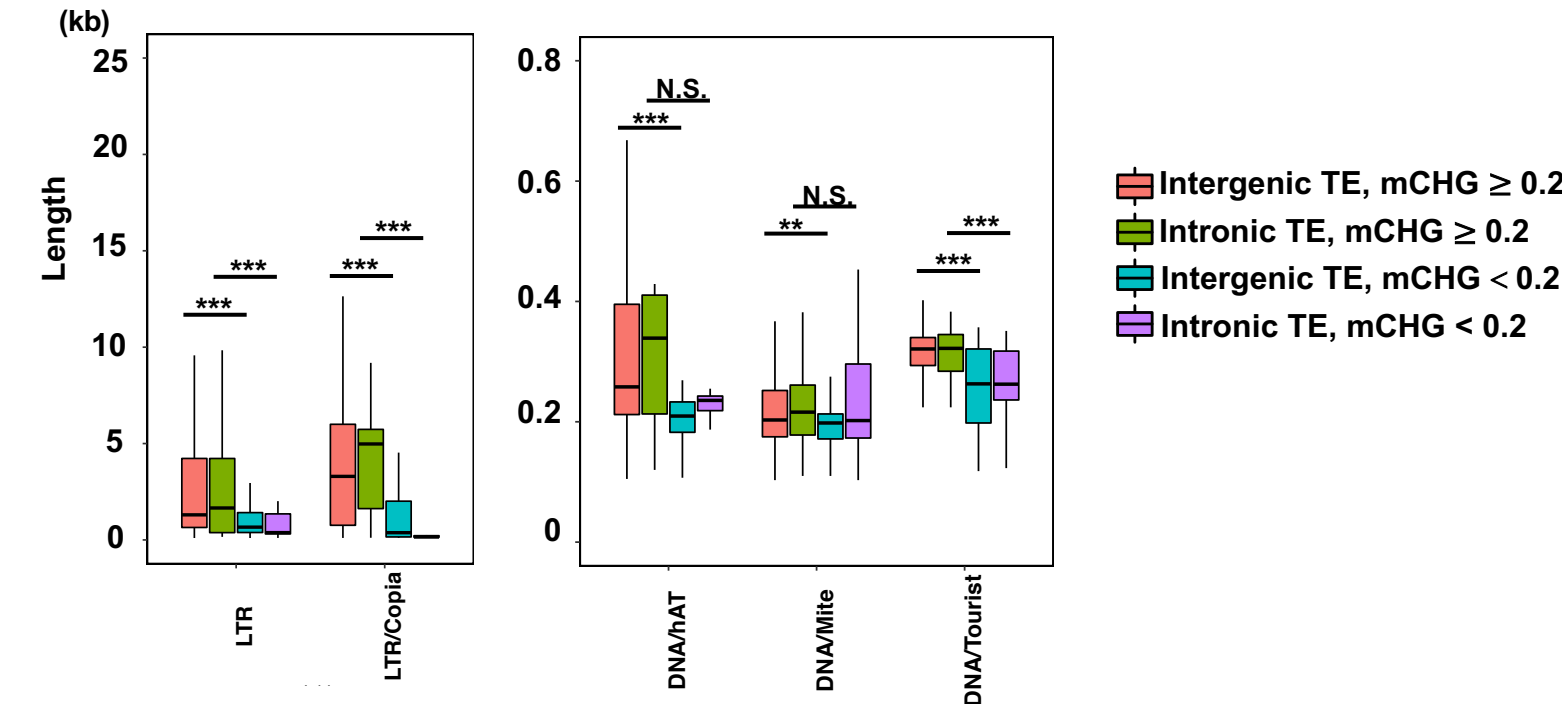

mCHH

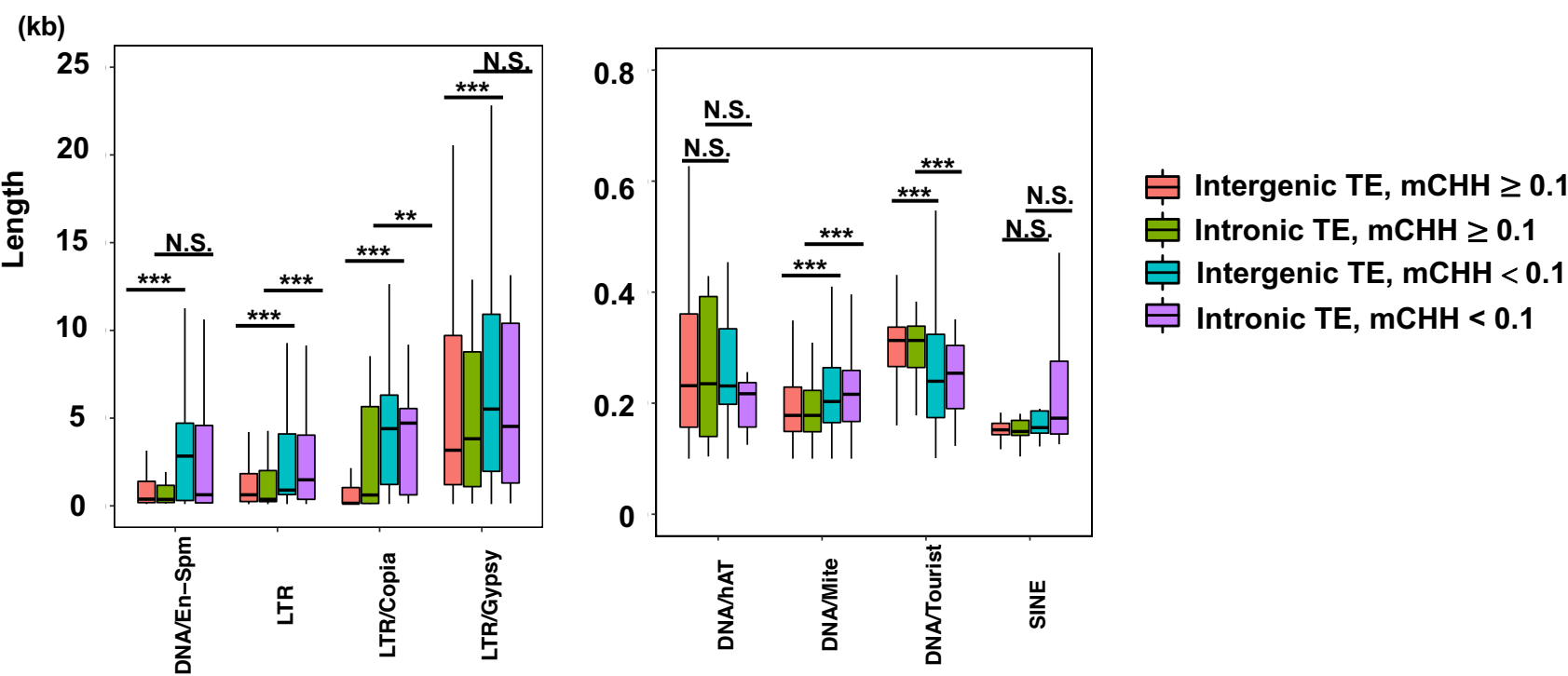

Supplement: S5 Fig — Boxplots showing length of representative intergenic and intronic TE families (>10 copies in each category) and their methylation levels in CG (high; mCG ≥ 0.9, low; mCG < 0.9), CHG (high; mCHG ≥ 0.2, low; mCHG < 0.2), and CHH (high; mCHH ≥ 0.1, low; mCHH < 0.1). * p < 0.05, ** p < 0.01, *** p < 0.001, Wilcoxon exact test. N.S.: no significance, p ≥ 0.05. TEs with methylation data at ≥ 5 Cs were analyzed. (PDF) [file pgen.1008637.s005.pdf]

**A**

**Intergenic MITE**

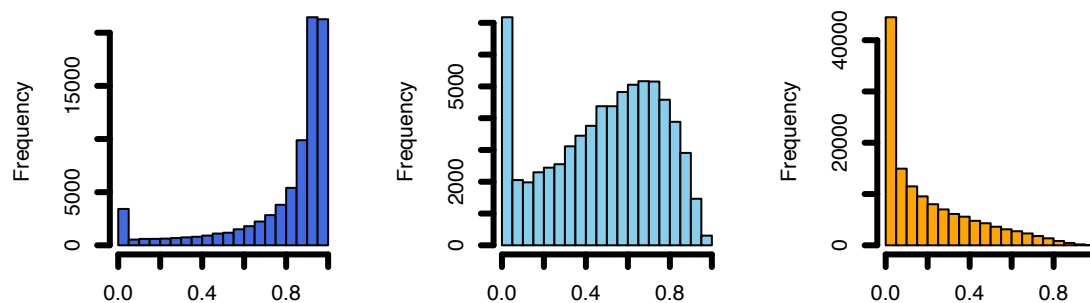

**Intronic MITE**

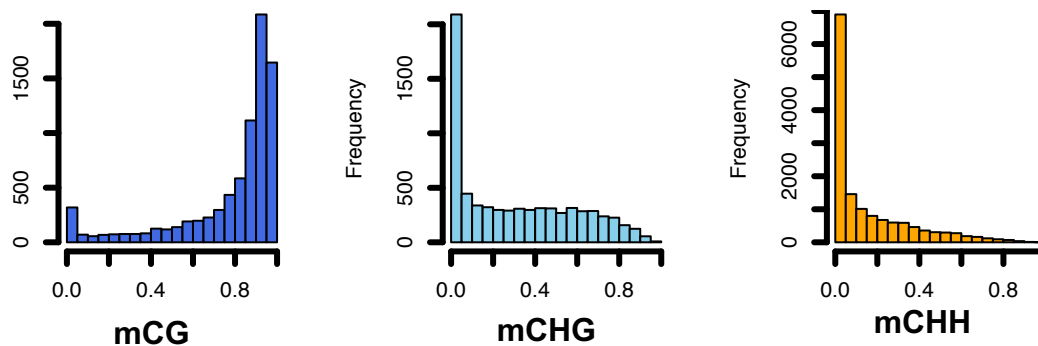

**B**

**Intergenic MITE**

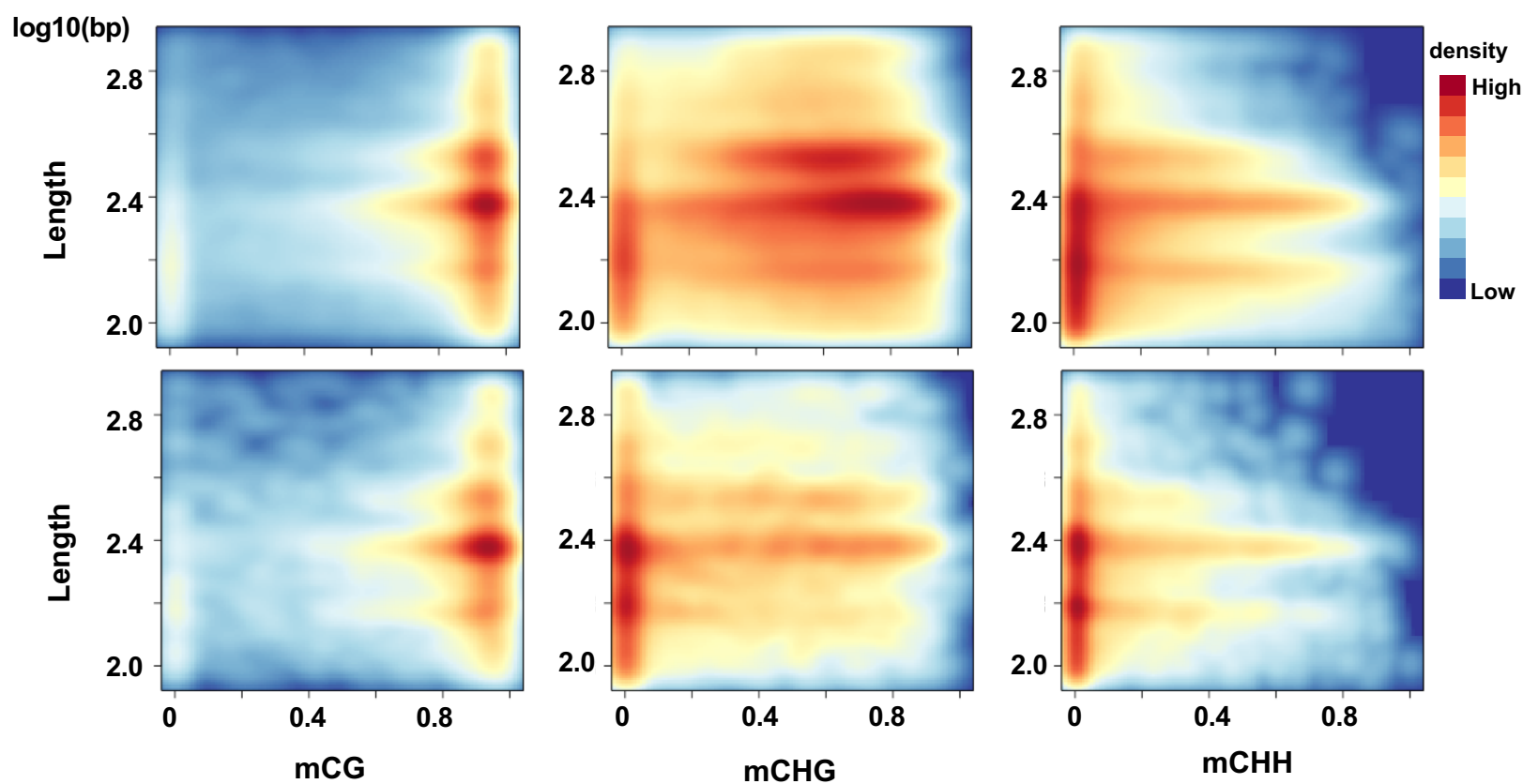

**Intronic MITE**

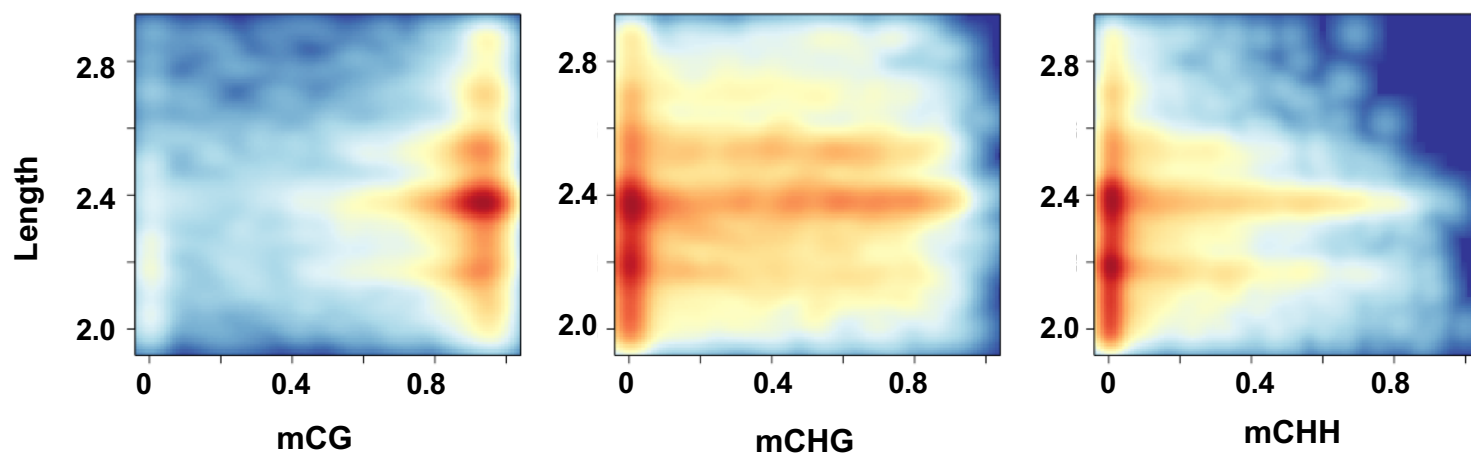

Supplement: S6 Fig — (A) Histograms of the number of representative intergenic and intronic MITEs (data retrieved from the P-MITE database [61] and their methylation levels (0 to 1) in CG, CHG, and CHH contexts. TEs with methylation data at ≥ 5 Cs were used in the analysis. (B) Density plots showing length (log10) and methylation levels (0 to 1) of intergenic and intronic MITEs in CG, CHG, and CHH contexts. (PDF) [file pgen.1008637.s006.pdf]

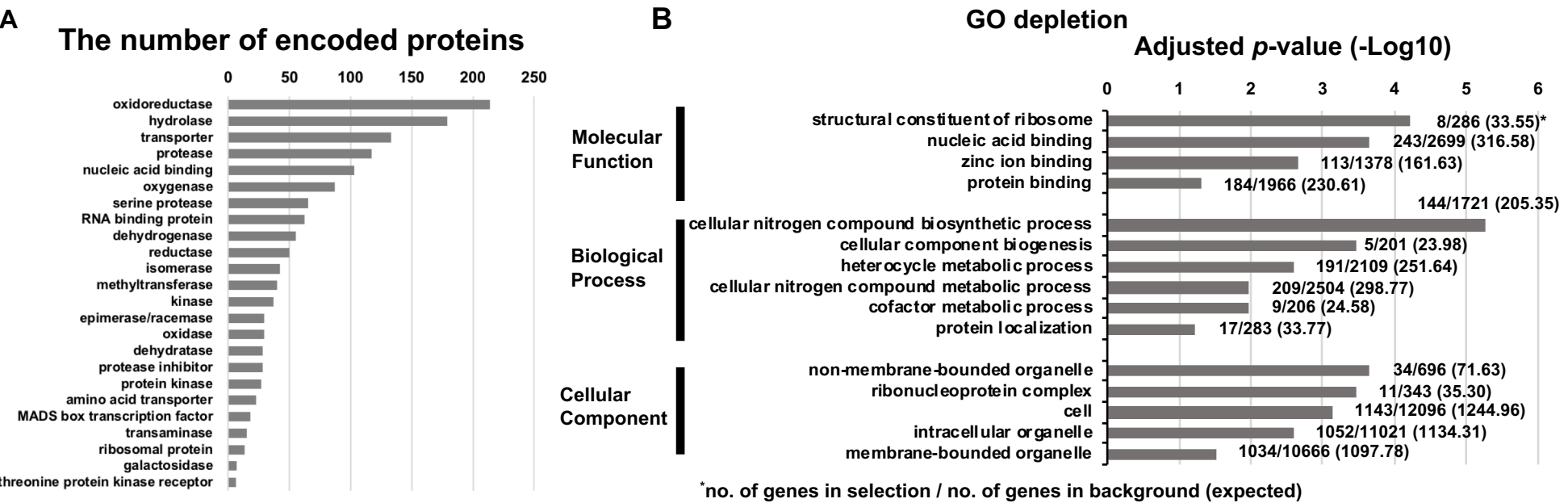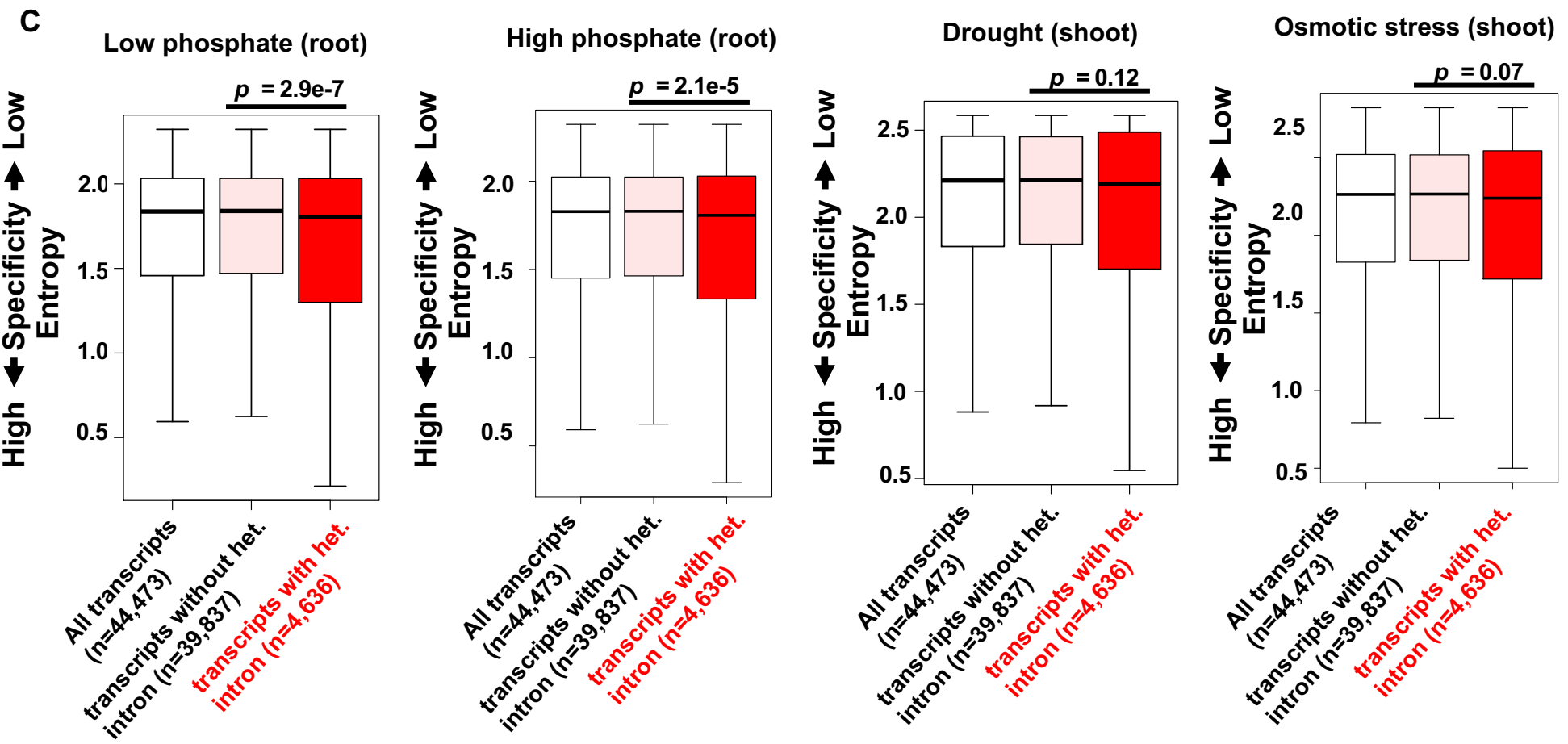

Supplement: S7 Fig — (A) Protein classes defined by the Panther database [119]. 1,407 of 4,227 genes containing heterochromatic introns matching the database are indicated. (B) Gene Ontology depletion for genes containing heterochromatic introns. P-values were obtained by Fisher test, and terms with FDR < 0.05 are indicated. (C) Expression changes of all genes and genes with or without heterochromatic introns by various stress treatments. Specificity of the responses to given treatments were measured as entropy values. P-values from Wilcoxon exact test are indicated. Effect size (r) in each analysis: Low phosphate; 0.024, High phosphate; 0.020, Drought; 0.007, Osmotic stress; 0.009. (PDF) [file pgen.1008637.s007.pdf]

A

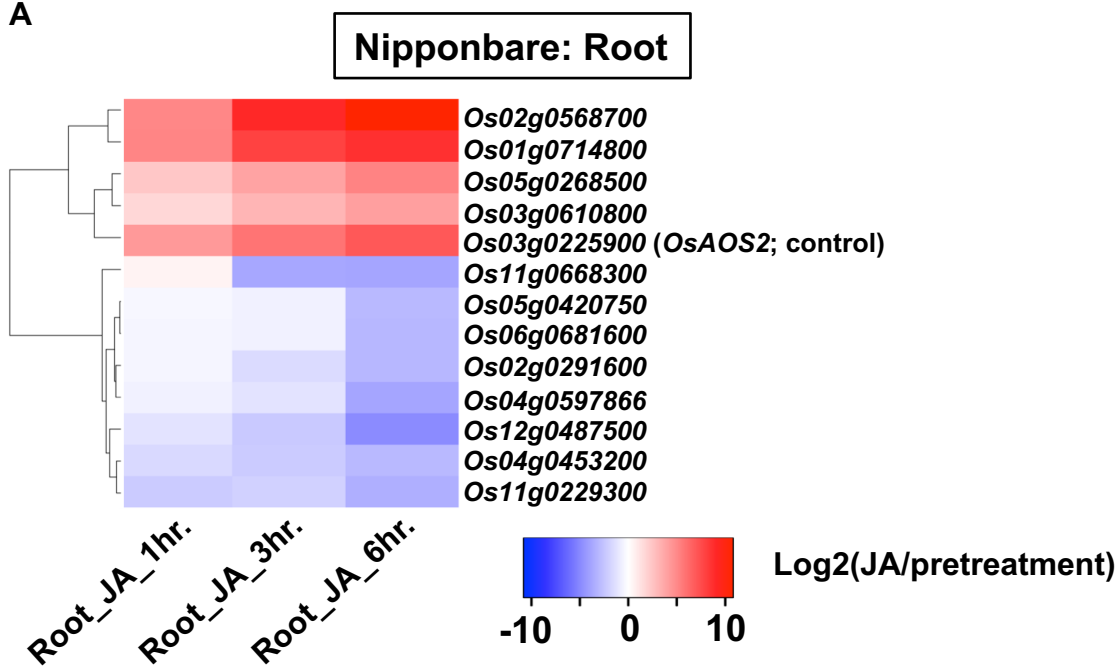

B

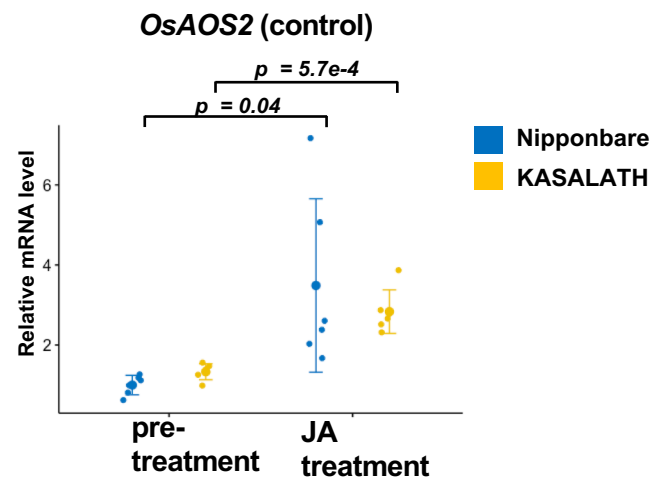

## Up-regulated genes

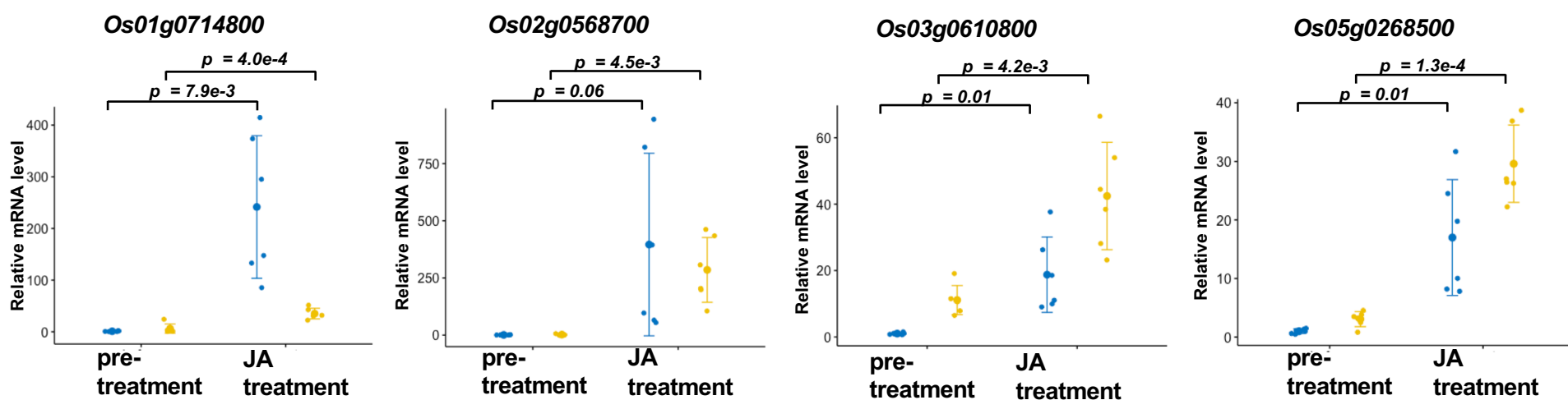

## Down-regulated genes

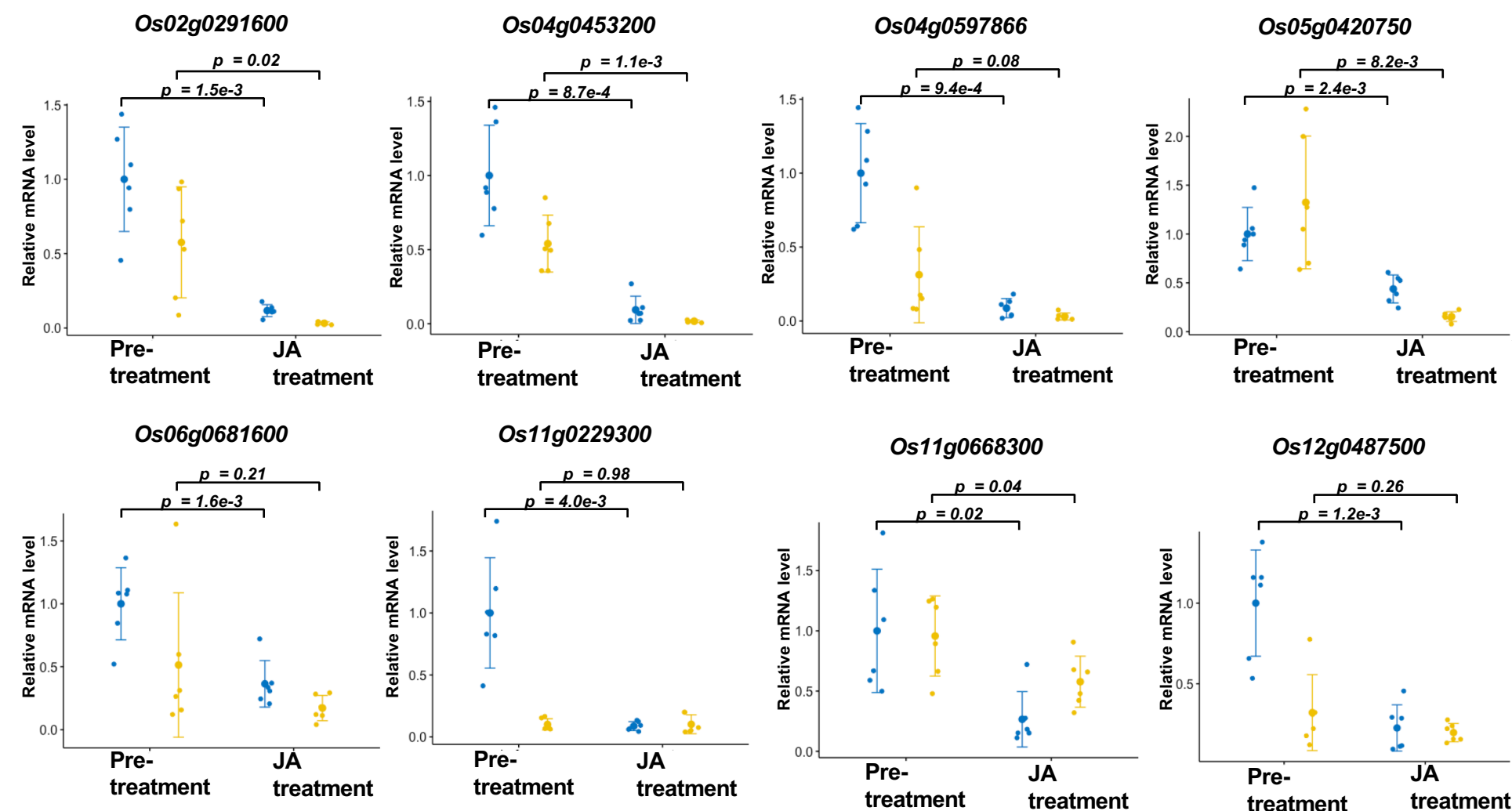

Supplement: S8 Fig — (A) Heatmap showing expression levels of the indicated genes after Jasmonic Acid (JA) treatment in the Nipponbare root. Expression data were obtained from TENOR [63]. (B) Quantitative RT-PCR (qRT-PCR) analysis of genes before (pre-treatment), and after JA (JA treatment). OsAOS2 was included as a control for JA-dependent induction of expression. Relative expression levels in each sample were normalized by UBQ1 expression levels, and the average of expression values in pre-treatment NB samples was set as 1, and plotted as dots (n = 6) with blue (NB) and yellow (KAS). The large dots and bars represent means of 6 biological replicates ± standard deviation (S. D.). P-values were obtained by t-test. (PDF) [file pgen.1008637.s008.pdf]

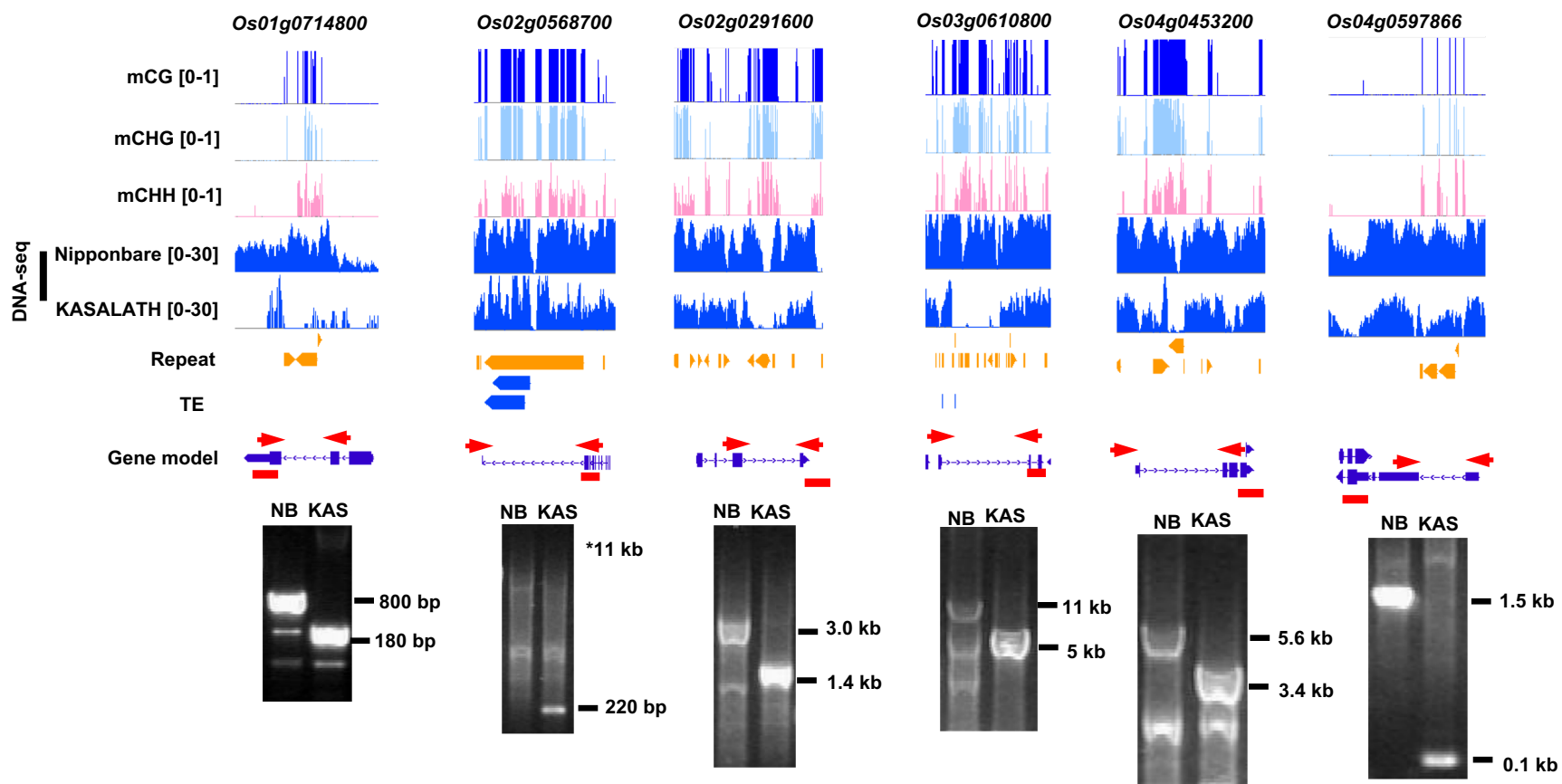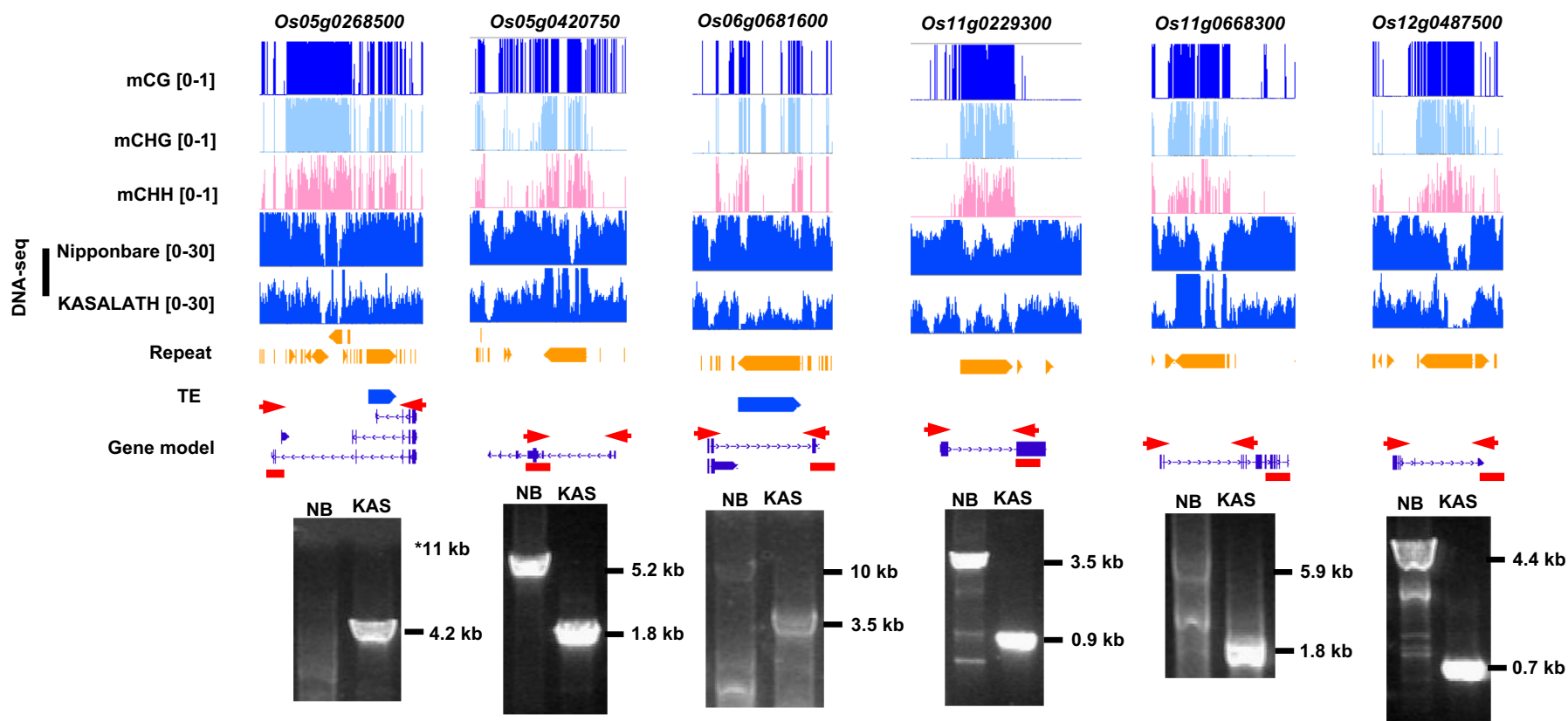

Supplement: S9 Fig — Insertion/deletion polymorphisms in Nipponbare and KASALATH. Tracks: Top to bottom: mCG ratio (0 to1), mCHG ratio (0 to1), mCHH ratio (0 to1), genome-resequencing data coverage (0 to 30) [65], repeats (orange), TE annotation (blue), gene model (purple). Structural variations detected by PCR are indicated under the tracks as gel pictures. Red arrows indicate the primer positions used for PCR amplifications shown in the gel panel. The region used for qRT-PCR is indicated as red bar. (PDF) [file pgen.1008637.s009.pdf]

A

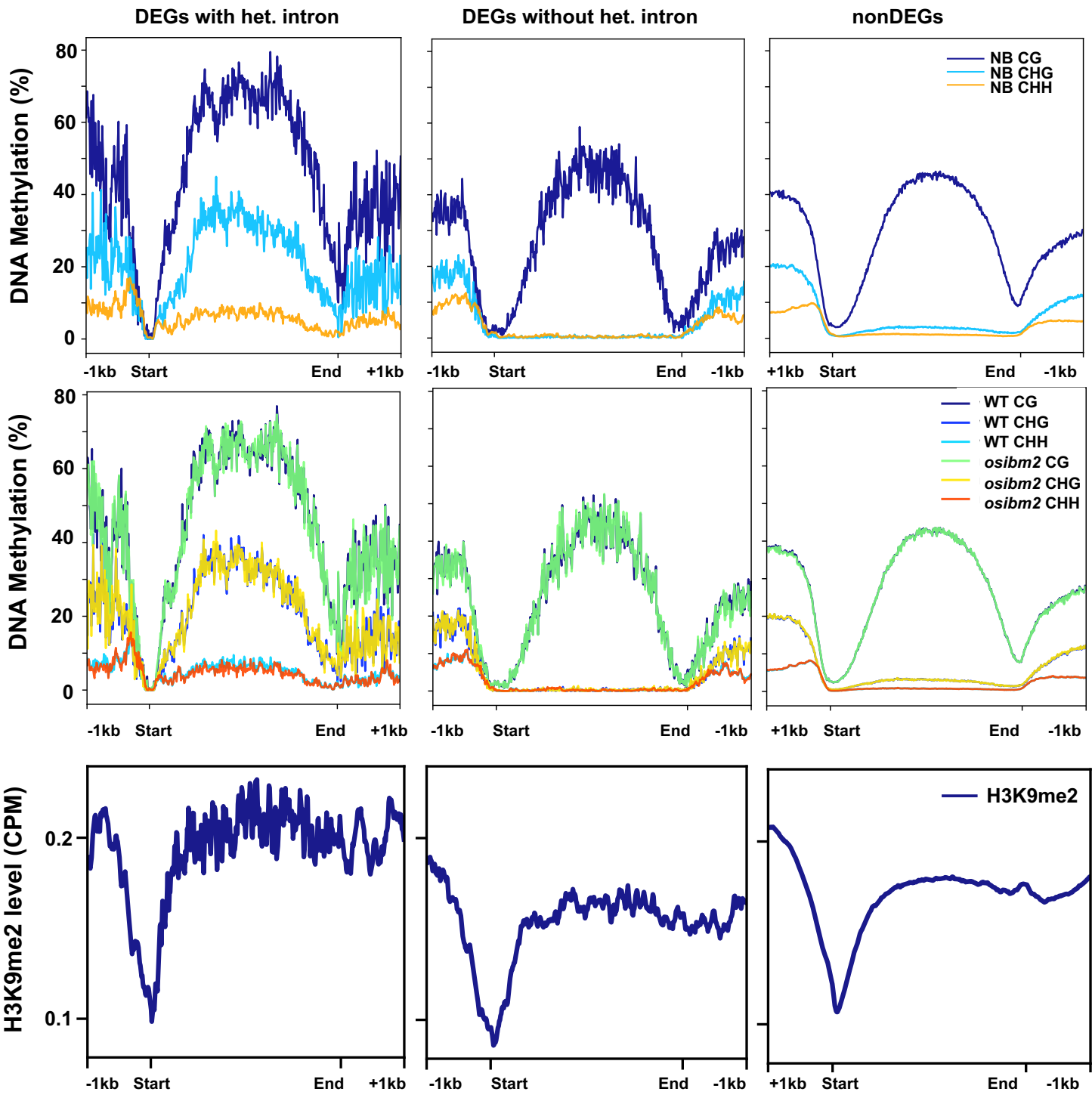

B

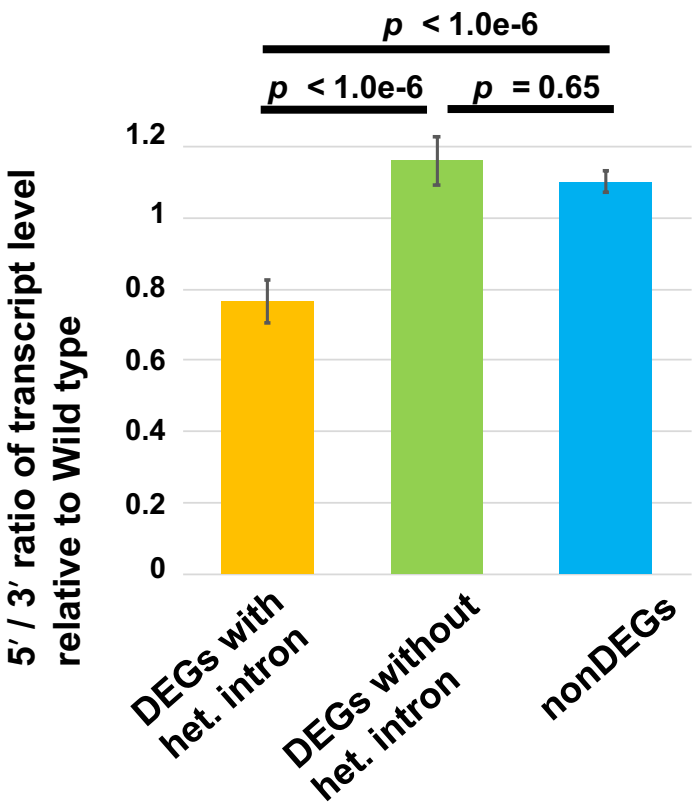

Supplement: S12 Fig — (A) (Top) DNA methylation levels of differentially expressed genes (DEGs) with heterochromatic introns (n = 93), DEGs without heterochromatic intron (n = 361), and non DEGs (n = 20293) in Nipponbare background. (middle) DNA methylation difference in osibm2 (osibm2_ g2 #24) and wild type at loci as above. (Bottom) H3K9 methylation levels at loci as above. (B) 5′/3′ ratio of transcripts mapped to up- and down-stream of introns relative to wild type. RNA-seq data from osibm2_ g2 #24 and WT (wild-type segregants of osibm2) were used. In each locus, the 5′/3′ ratio of a representative transcript variant with TPM >1 was used for calculation. Bars represent the means of DEGs with heterochromatic introns (n = 68), DEGs without heterochromatic intron (n = 335), and randomly selected 300 nonDEG loci ± S.E.M. p-values were obtained by Tukey-Kramer test. (PDF) [file pgen.1008637.s012.pdf]

A

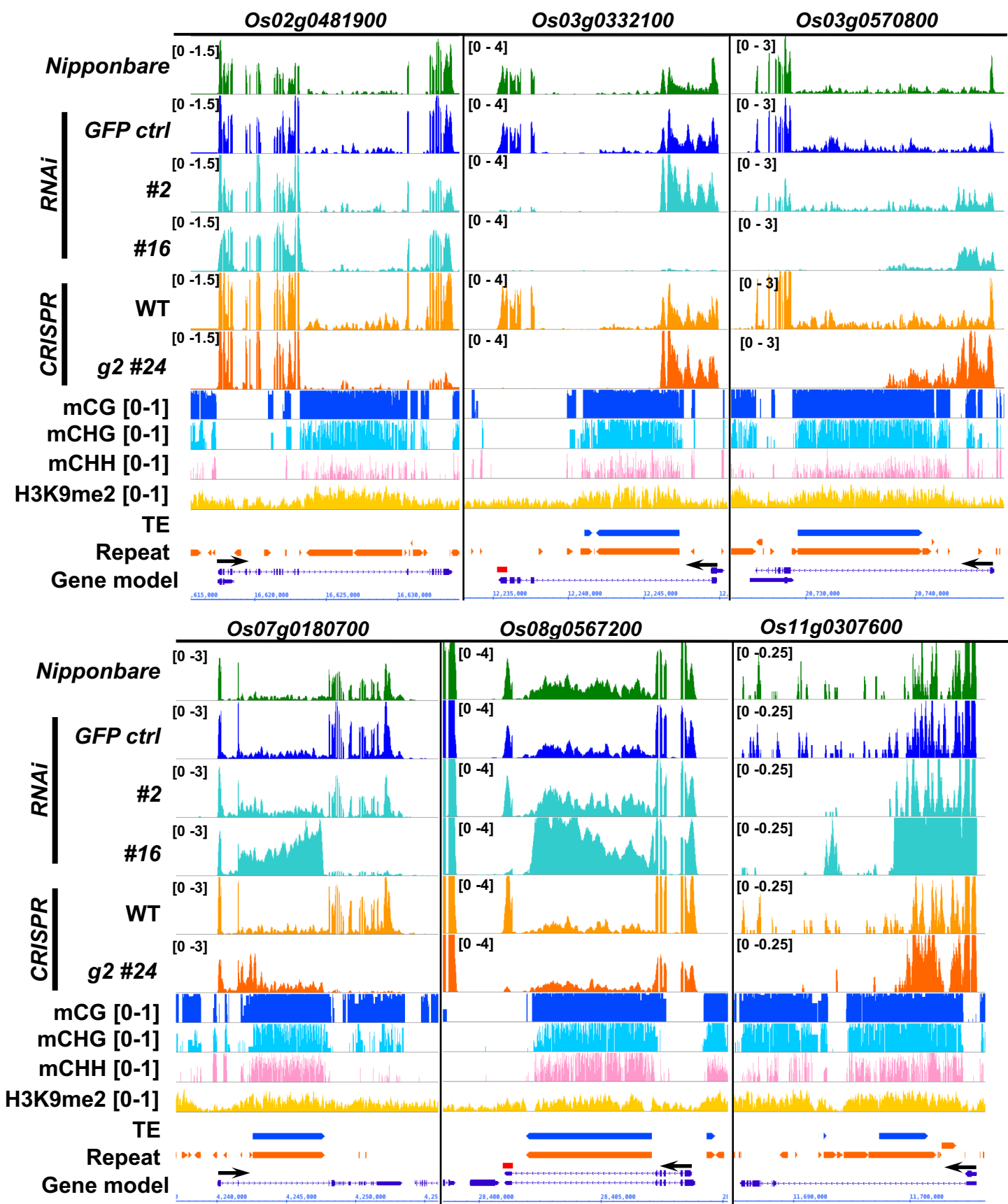

B

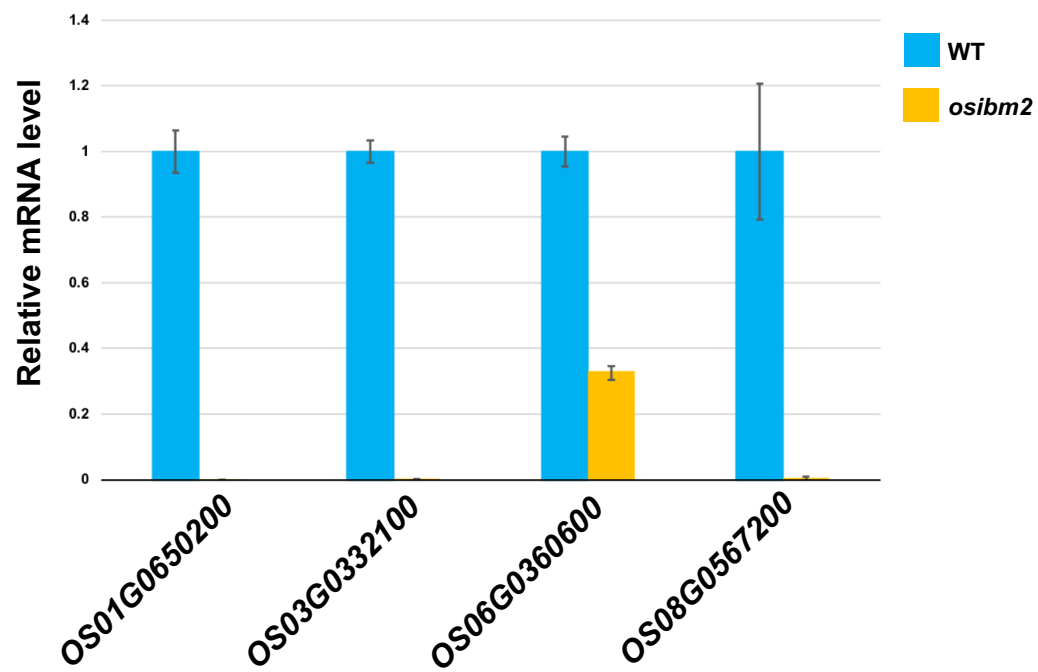

Supplement: S13 Fig — (A) Representative rice genome loci showing altered expression patterns in mutants of OsIBM2. Tracks; Top to bottom: RNAseq (Reads per Million are indicated at top left), mCG ratio (0 to1), mCHG ratio (0 to1), mCHH ratio (0 to1), H3K9me2 (RPM; 0 to 1), TE annotation (blue), repeats (orange), gene model (purple). The black arrow indicates the orientation of coding sequence. (B) Quantitative RT-PCR (qRT-PCR) analysis of expression of genes containing heterochromatic introns in osibm2_g2#24 (osibm2) and WT (wild-type segregants of osibm2). Primer positions are indicated in Fig 5F and S13A Fig as red bars. Expression levels in each sample were normalized by UBQ1 expression levels, and the average of OsIBM2/UBQ1 in WT was set as 1. Bars represent the means of three biological replicates ± S. D. (n = 3). (PDF) [file pgen.1008637.s013.pdf]

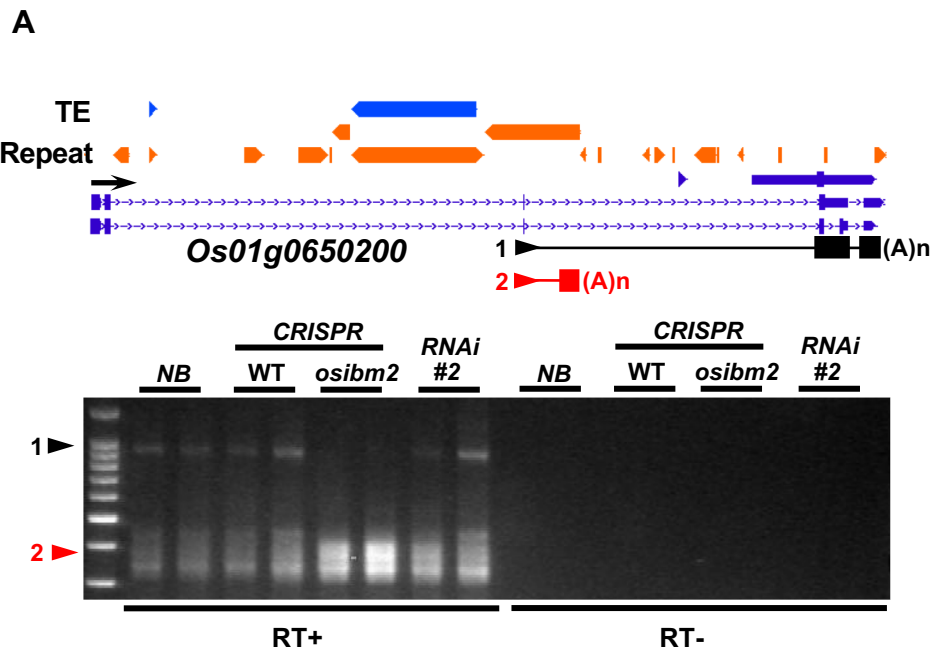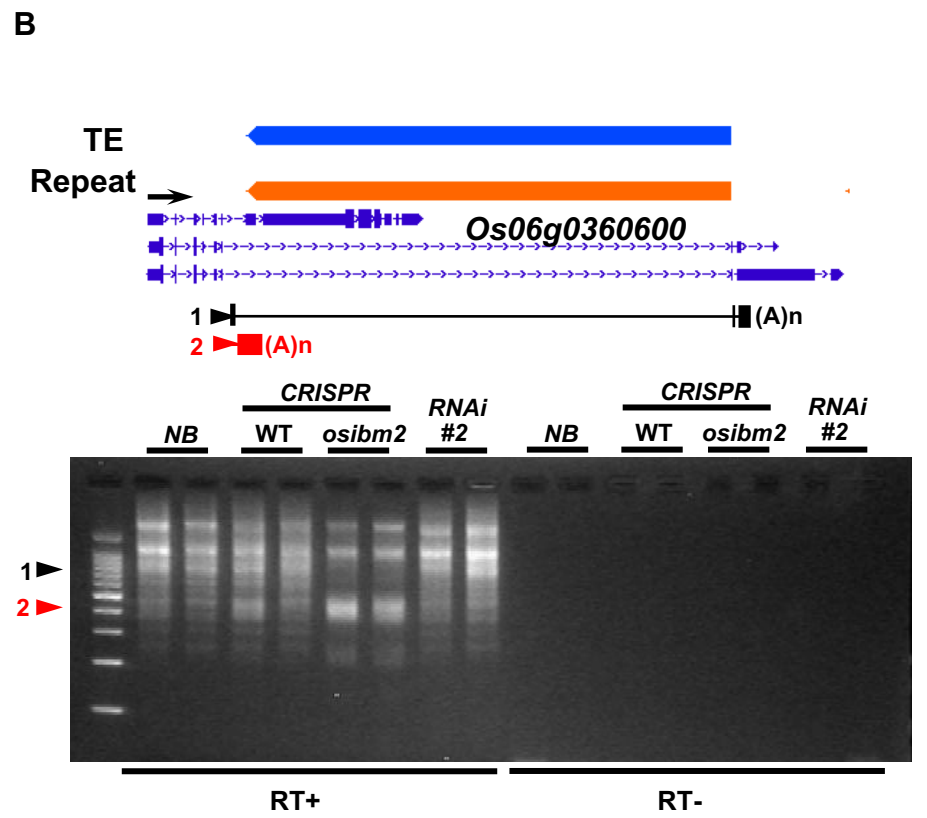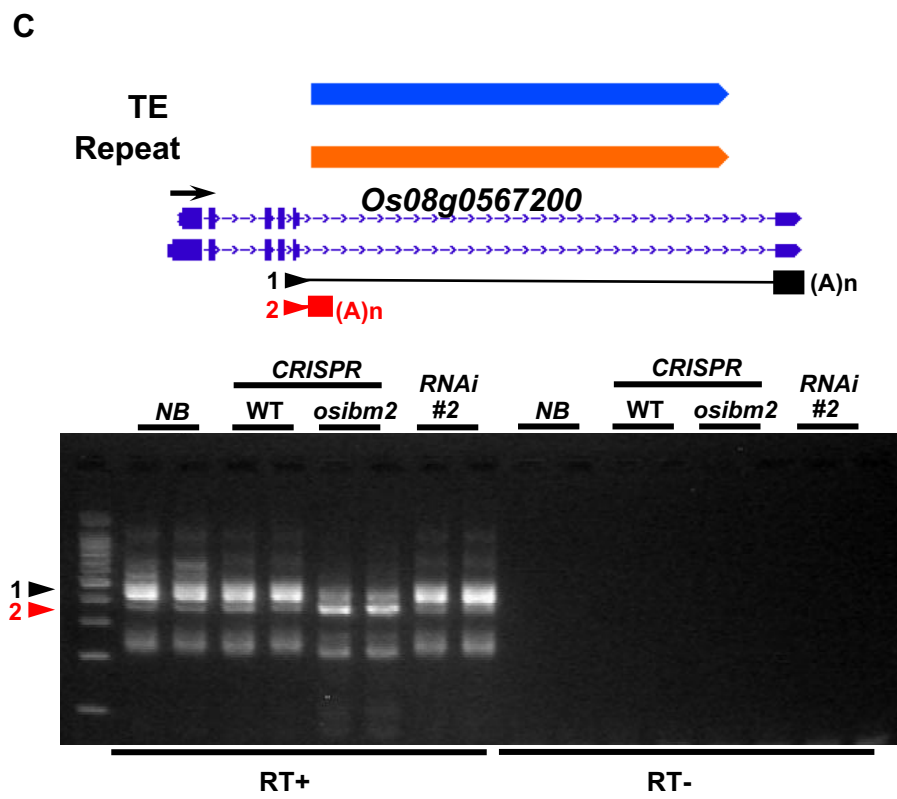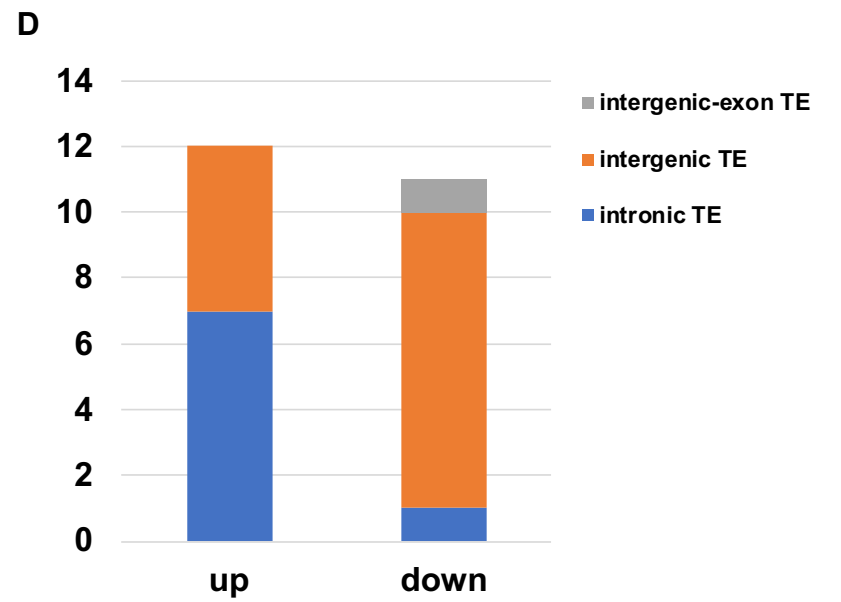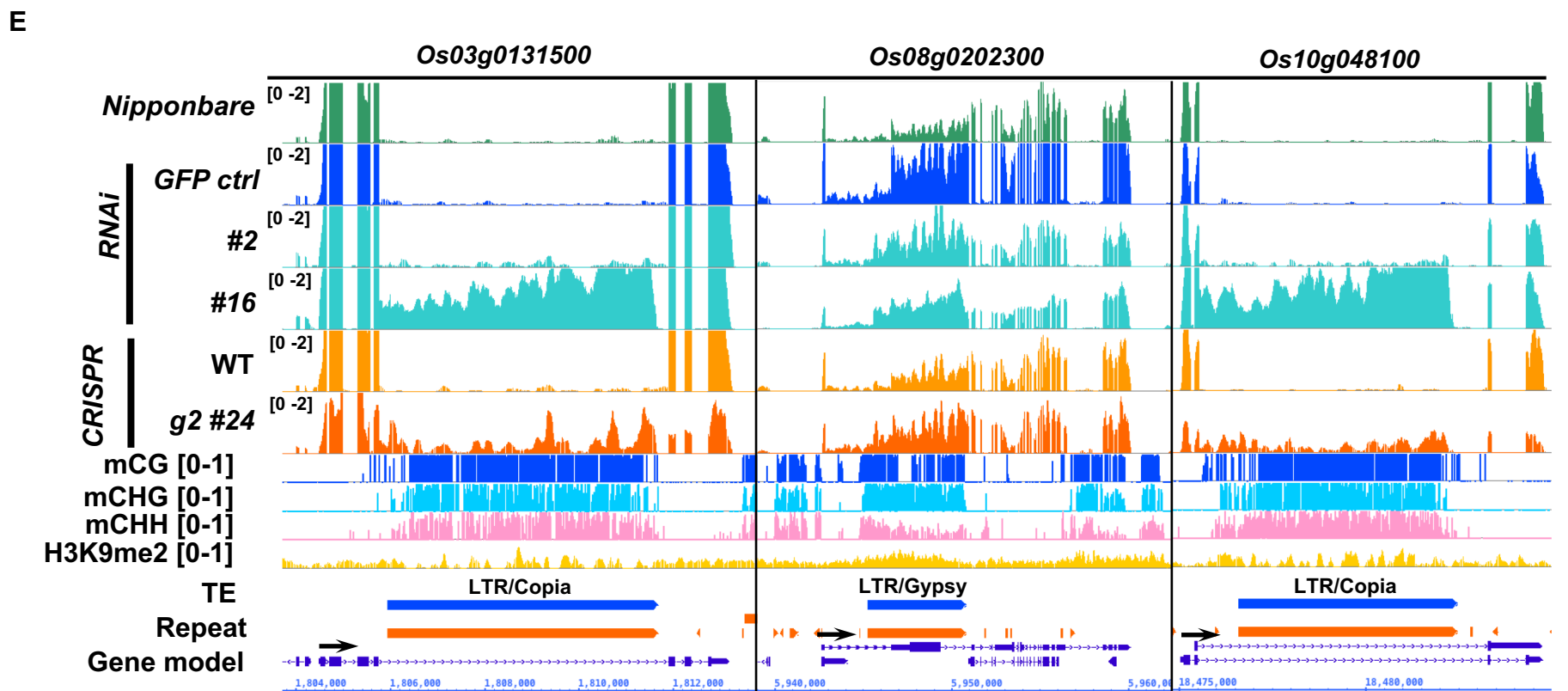

Supplement: S14 Fig — (A) 3′ RACE of Os01g0650200. Upper panel: Structure of Os01g0650200 locus and polyadenylated mRNA variants detected by 3′ RACE. Exons and spliced introns confirmed by sequencing analysis are shown as black/red boxes and lines, respectively. Primer positions used for 3′ RACE are indicated by arrowheads. Lower panel: Gel picture of DNA fragments amplified by 3′ RACE. Two biological replicates for each genotype were examined. DNA fragments indicated by arrowheads were cloned and sequenced at least for 8 clones, and the representative sequences supported with more than 3 clones are shown in the upper panel. The black arrow indicates the orientation of coding sequence. NB: Nipponbare; osibm2: osibm2_g2#24; WT: wild-type segregants of osibm2; (A)n: polyadenylation. (B) 3′ RACE of Os06g0360600 as in (A). (C) 3′ RACE of Os08g0567200 as in (A). (D) The number of TEs showing expression changes in osibm2_g2#24 (osibm2). 22 LTR TEs, and 1DNA/En-Spm showed significant changes (q<0.05) of both up-regulation (12 TEs) and down-regulation (11 TEs). (E) Rice genome loci showing altered expression patterns of intronic TEs in mutants of OsIBM2. Tracks; Top to bottom: RNAseq (Reads per Million are indicated at top left), mCG ratio (0 to1), mCHG ratio (0 to1), mCHH ratio (0 to1), H3K9me2 (RPM; 0 to 1), TE annotation (blue), repeats (orange), gene model (purple). The black arrow indicates the orientation of coding sequence. (PDF) [file pgen.1008637.s014.pdf]

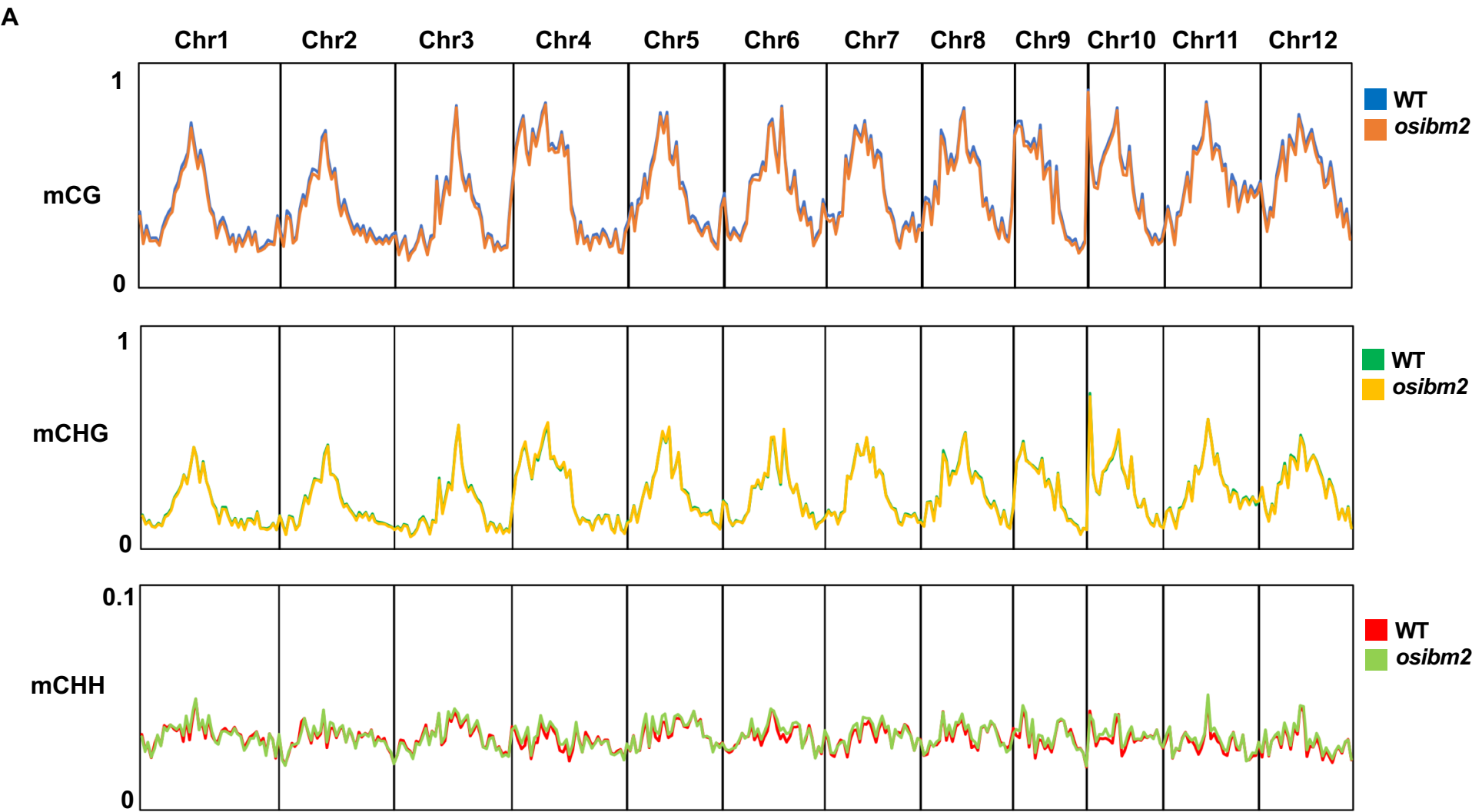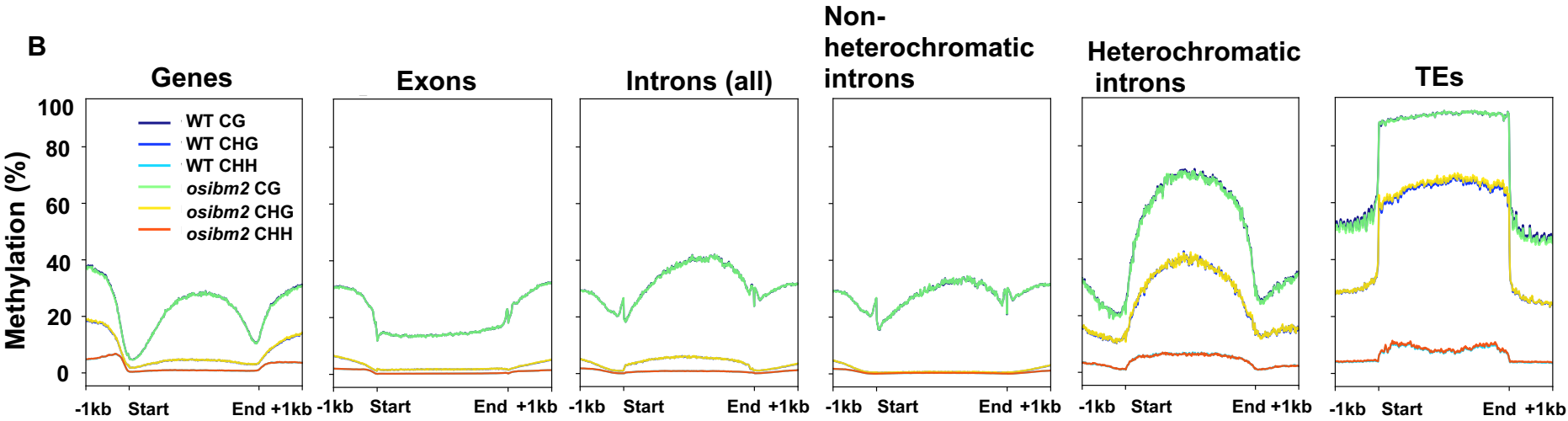

Supplement: S15 Fig — (A) Genome-wide DNA methylation in osibm2_g2#24 (osibm2, T4) and their wild type segregating siblings (WT, T4) in CG, CHG and CHH contexts for each chromosome. Average methylation levels in 1 MB bins were plotted. (B) Metaplots of DNA methylation in osibm2_g2#24 (osibm2) and their wild-type segregating siblings (WT) in CG, CHG and CHH contexts for indicated genome features. (PDF) [file pgen.1008637.s015.pdf]

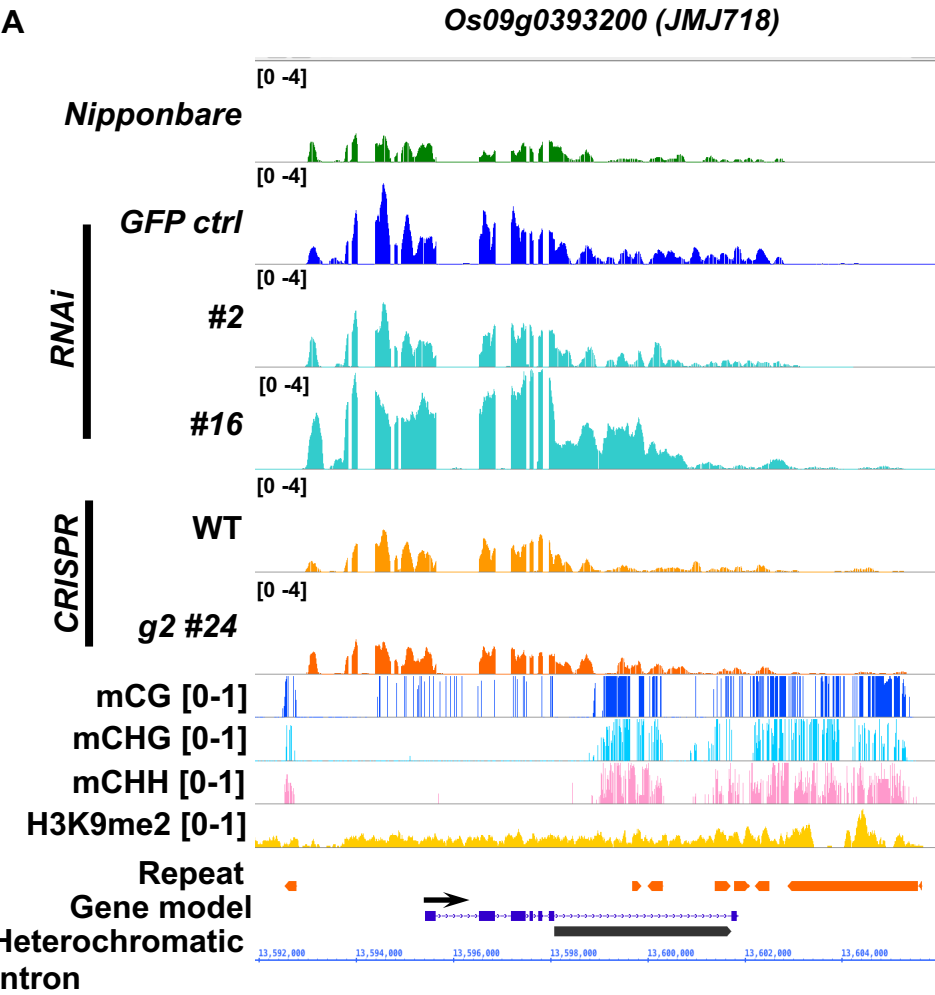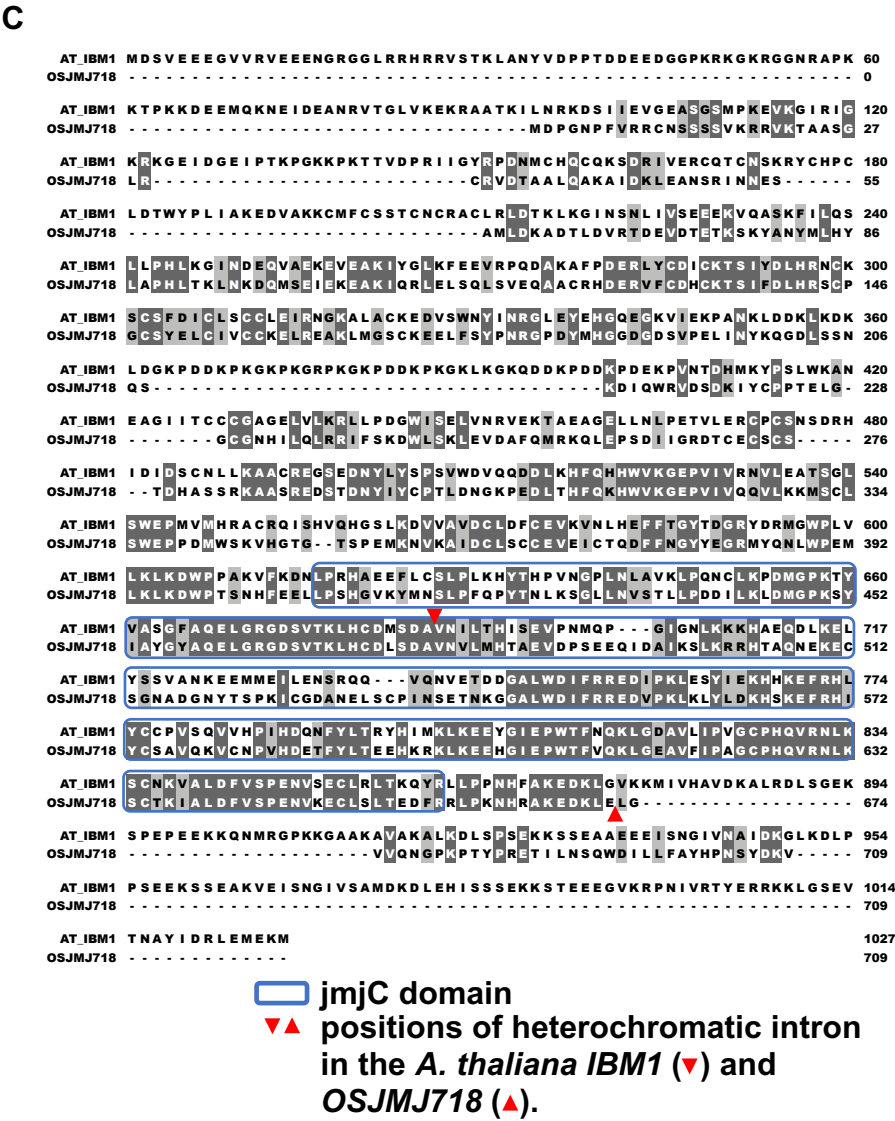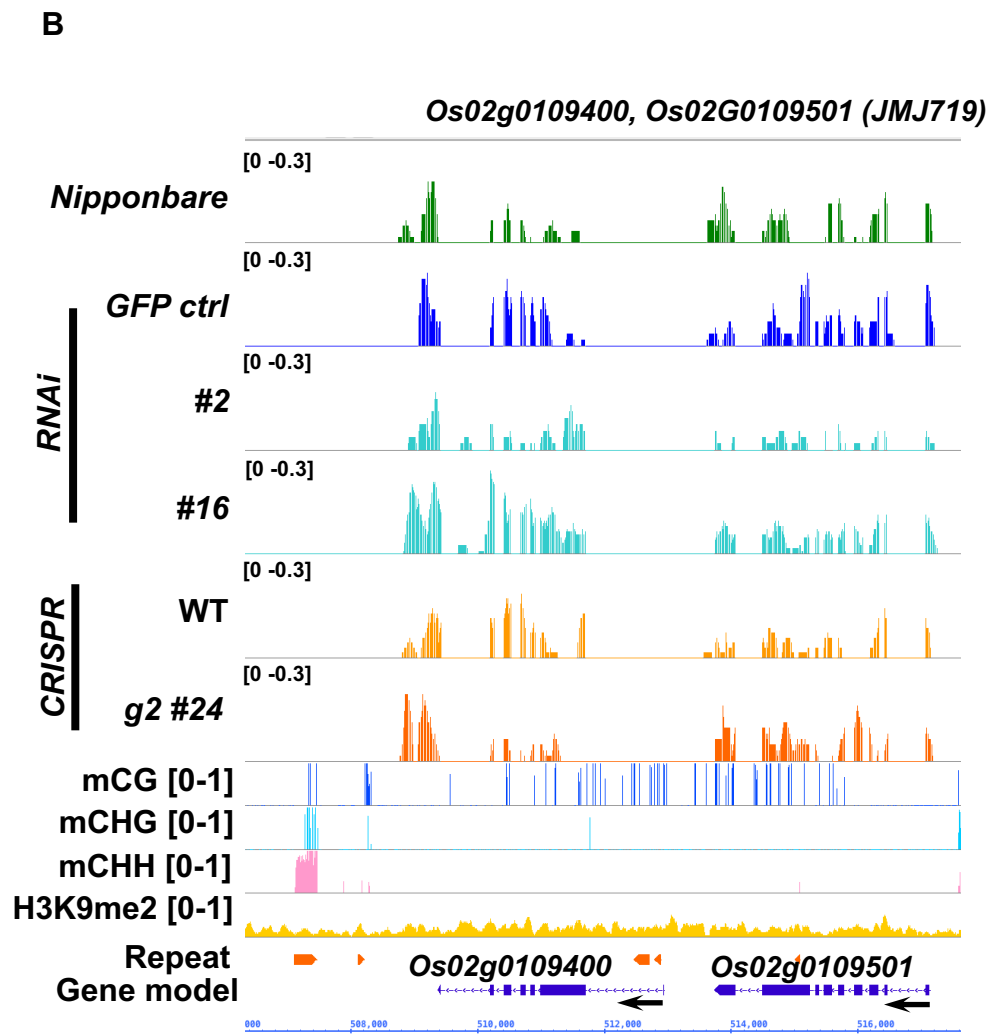

Supplement: S16 Fig — Genome loci for OsJMJ718 (Os09g0393200) (A) and OsJMJ719 (Os02g0109400, Os02G0109501) (B). RNA-seq, DNA methylation and H3K9me2 tracks are shown as in S13 Fig. (C) An alignment of amino acids sequences of A. thaliana IBM1 (At_IBM1) and OsJMJ718. The amino acid sequence of the N-terminal part of OsJMJ718 is predicted based on RNA-seq reads in this study. The alignment was generated by CLUSTAL W [131]. Jumonji-C (JmjC) domains predicted by SMART [132] are circled with blue lines. Positions of heterochromatic introns are indicated by red arrowheads. (PDF) [file pgen.1008637.s016.pdf]
