# Supplementary material for: Transcriptional regulation of genes bearing intronic heterochromatin in the rice genome
Source: PLoS Genet. 2020 Mar 18;16(3):e1008637. doi: 10.1371/journal.pgen.1008637 (PMC7145194; doi:10.1371/journal.pgen.1008637)
Supplement: S10 Fig — Amino acid alignment of homologs of OsIBM2 in plants based on [34]. Bromo-Adjacent Homology (BAH) domain and RNA-Recognition Motif (RRM) are framed with a blue line. Arrows indicate regions designed for guide RNAs used for CRISPR-Cas9 mediated deletion. At; Arabidopsis thaliana: Zm; Zea mays: Os; Oryza sativa: Sb; Sorghum bicolor: Pt; Populus trichocarpa: Rc; Ricinus communis. (PDF) [file pgen.1008637.s010.pdf]

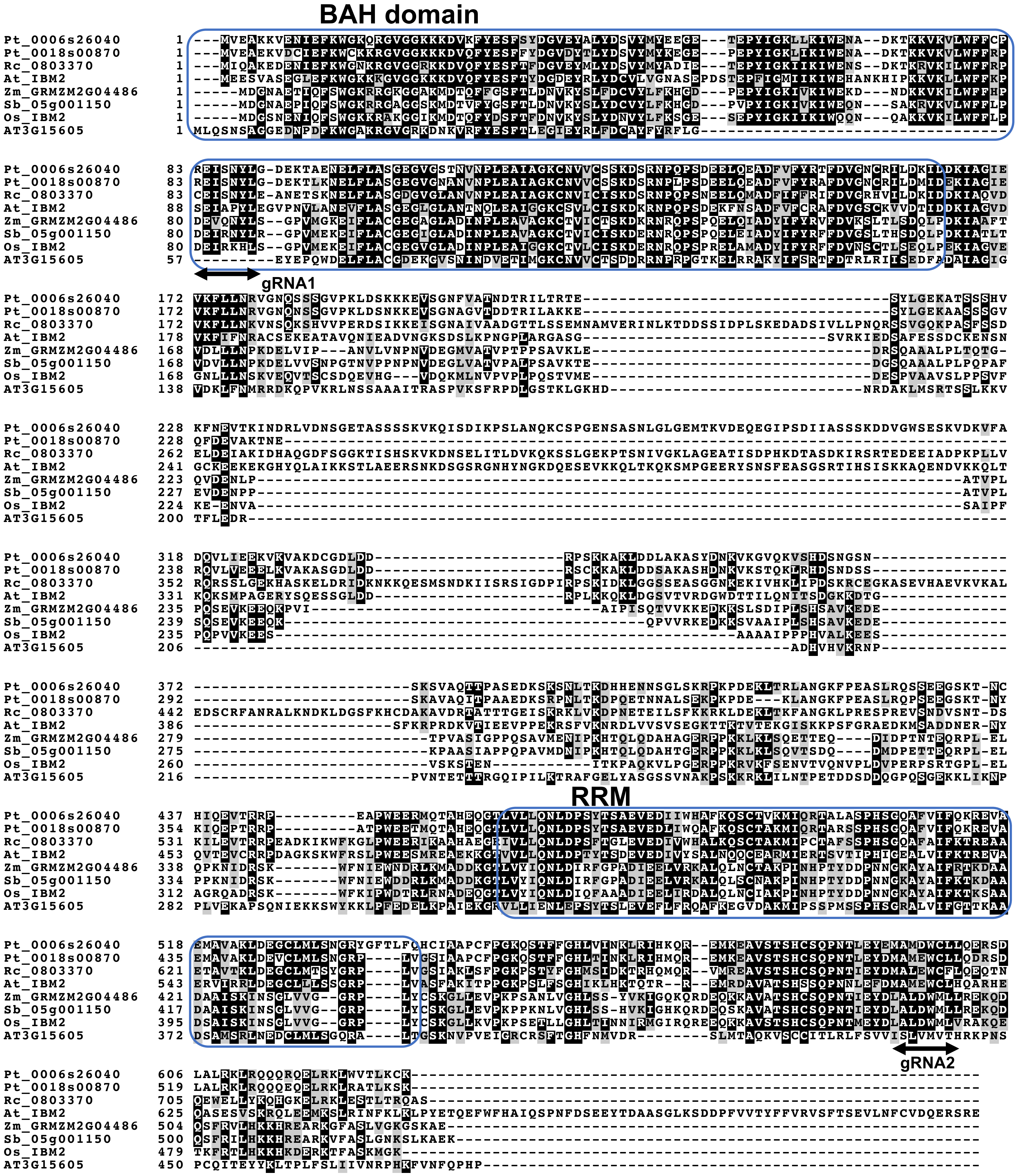

BAH domain
Pt_0006s26040 1 ---MVEAKKVENIEFKWGKQRGVGGKKKDVKFYESFSYDGVEYALYDSVYMYEEGE---TEPYIGKLLKIWENA--DKTKKVKVLWFFCP
Pt_0018s00870 1 ---MVEAEKVDCIEFKWCKKRGVGGKKKDVQFYESFFYDGVDYTLYDSVYMYKEGE---PEPYIGKLIKIWENA--DKTKKVKVLWFFRP
Rc_0803370 1 ---MIQAKEDENIEFKWGNKRGVGGRKKDVQFYESFTFDGVEYMLYDSVYMYADIE---TEPYIGKIIKIWENS--DKTKRVKILWFFRP
At_IBM2 1 ---MEESVASEGLEFKWGKKKGVGGKKKDVQFYESFTYDGDEYRLYDCVLVGNASEPDSTEPFIGMIIKIWEHANKHIPKKVKLLWFFKP
Zm_GRMZM2G04486 1 -----MDGNAETIQFSWGKRRGKGGAKMDTQFFGSFTLDNVKYSLFDCVYLFKHGD---PEPYIGKIVKIWEKD---NDKKVKILWFFHP
Sb_05g001150 1 -----MDGNAEPIQFSWGKRRGAGGSKMDTVFYGSFTLDNVKYSLYDCVYLFKHGD---PVPYIGKIVKIWEQN---SAKRVKVLWFFLP
Os_IBM2 1 -----MDGSNENIQFSWGKKRAKGGIKMDTQFYDSFTFDNVKYSLYDNVYLFKSGE---SEPYIGKIIKIWQQN---QAKKVKILWFFLP
AT3G15605 1 MLQSNSAGGEDNPDFKWGAKRGVGRKDNKVRFYESFTLEGIEYRLFDCAYFYRFLG----------------------------------
Pt_0006s26040 83 REISNYLG-DEKTAENELFLASGEGVGSTNVNPLEAIAGKCNVVCSSKDSRNPQPSDEELQEADFVFYRTFDVGNCRILDKIDDKIAGIE
Pt_0018s00870 83 REISNYLG-DEKTLKNELFLASGEGVGNANVNPLEAIAGKCNVVCSSKDSRNPLPSDEELQEADFVFYRAFDVGNCRILDMIDEKIAGIE
Rc_0803370 83 CEISNYLE-ANETSKNELFLASGDGVGLANVNPLEAIAGKCNVICISKDSRNPQPSNEELQMADFIFFRIFDVGRHVILDKIDDKIAQVD
At_IBM2 88 SEIAPYLEGVPNVLANEVFLASGEGLGLANTNQLEAIGGKCSVLCISKDKRNPQPSDEKFNSADFVFCRAFDVGSCKVVDTIDDKIAGVD
Zm_GRMZM2G04486 80 DEVQNYLS--GPVMGKEIFLACGEGAGLADINPLEAVAGKCTVICTSKDKRNRQPSPQELQIADYIFYRVFDVKSLTLSDQLPDKIAAFT
Sb_05g001150 80 DEIRNYLR--GPVMEKEIFLACGEGIGLADINPLEAVAGKCTVICISKDERNRQPSPQELEIADYIFYRFFDVGSLTHSDQLPDKIATLT
Os_IBM2 80 DEIRKHLS--GPVMEKEIFLACGEGVGLADINPLEAIGGKCTVLCISKDERNRQPSPRELAMADYIFYRFFDVNSCTLSEQLPEKIAGVE
AT3G15605 57 ---------EYEPQWDELFLACGDEKGVSNINDVETIMGKCNVVCTSDDRRNPRPGTKELRRAKYIFSRTFDTRLRIISEDFADAIAGIG
gRNA1
Pt_0006s26040 172 VKFLLNRVGNQSSSGVPKLDSKKKEVSGNFVATNDTRILTRTE---------------------------------------SYLGEKATSSSHV
Pt_0018s00870 172 VKFLLNRVGNQNSSGVPKLDSNKKEVSGNAGVTDDTRILAKKE---------------------------------------SYLGEKAASSSGV
Rc_0803370 172 VKFLLNKVNSQKSHVVPERDSIKKEISGNAIVAADGTTLSSEMNAMVERINLKTDDSSIDPLSKEDADSIVLLPNQRSSVGQKPASFSSD
At_IBM2 178 VKFIFNRACSEKEATAVQNIEADVNGKSDSLKPNGPLARGASG----------------------------SVRKIEDSAFESSDCKENSN
Zm_GRMZM2G04486 168 VDLLLNPKDELVIP---ANVLVNPNVDEGMVATVPTPPSAVKLE--------------------------------------DRSQAAALPLTQTG-
Sb_05g001150 168 VDVLLNPKDELVVSNPGTNVPPNPNVDEGLVATVPALPSAVKTE--------------------------------------DGSQAAALPLPQPAF
Os_IBM2 168 GNLLLNSKVEQVTSCSDQEVHG---VDQKMLNVPVPLPQSTVME--------------------------------------DESPVAAVSLPPSVF
AT3G15605 138 VDKLFNMRRDKQPVKRLNSSAAAITRASPVKSFRPDLGSTKLGKHD--------------------------------NRDAKLMSRTSSLKKV
Pt_0006s26040 228 KFNEVTKINDRLVDNSGETASSSSKVKQISDIKPSLANQKCSPGENSASNLGLGEMTKVDEQEGIPSDIIASSSKDDVGWSESKVDKVFA
Pt_0018s00870 228 QFDEVAKTNE--
Rc_0803370 262 ELDEIAKIDHAQGDFSGGKTISHSKVKDNSELITLDVKQKSSLGEKPTSNIVGKLAGEATISDPHKDTASDKIRSRTEDEEIADPKPLLV
At_IBM2 241 GCKEEEKEKGHYQLAIKKSTLAEERSNKDSGSRGNHYNGKDQESEVKKQLTKQKSMPGEERYSNSFEASGSRTIHSISKKAQENDVKKQLT
Zm_GRMZM2G04486 223 QVDENLP--ATVPL
Sb_05g001150 227 EVDENPP--ATVPL
Os_IBM2 224 KE-ENVA--SAIPF
AT3G15605 200 TFLEDR--
Pt_0006s26040 318 DQVLIEEKVKVAKDCGDLDD-----------------------RPSKKAKLDDLAKASYDNKVKGVQKVSHDSNGSN--------------
Pt_0018s00870 238 RQVLVEEELKVAKASGDLDD-----------------------RSCKKAKLDDSAKASHDNKVKSTQKLRHDSNDSS--------------
Rc_0803370 352 RQRSSLGEKHASKELDRIDKNKKQESMSNDKIISRSIGDPIRPSKIDKLGGSSEASGGNKEKIVHKLIPDSKRCEGKASEVHAEVKVKAL
At_IBM2 331 KQKSMPAGERYSQESSGLDD-----------------------RPLKKQKLDGSVTVRDGWDTTILQNITSDGKKDTG--------------
Zm_GRMZM2G04486 235 PQSEVKEEQKPVI-----------------------------AIPISQTVVKKEDKKSLSDIPLSHSAVKEDE--------------
Sb_05g001150 239 SQSEVKEEQK---QPVVRKEDKKSVAAIPLSHSAVKEDE--------------
Os_IBM2 235 PQPVVKEES--AAAAIPPPHVALKEES--------------
AT3G15605 206 --ADHVHVKRNP--------------
Pt_0006s26040 372 -----------------------SKSVAQTTPASEDKSKSNLTKDHHENNSGLSKRPKPDEKLTRLANGKFPEASLRQSSEEGSKT-NC
Pt_0018s00870 292 -----------------------SKAVAQITPAAEDKSRPNLTKDPQETNNALSEKPKPDE----KLANGKFPEASLRQPSEEGSKT-NY
Rc_0803370 442 EDSCRFANRALKNDKLDGSFKHCDAKAVDRTATTTGEISKRKLVKDPNETEILSFKKRKLDEKLTKFANGKLPRESPREVSNDVSNT-DS
At_IBM2 386 ----------------------SFKRPRDKVTIEEVPPEKRSFVKNRDLVVSVSEGKTTKTVTEKGISKKPSFGRAEDKMSADDNER-NY
Zm_GRMZM2G04486 279 -------------------------TPVASIGPPQSAVMENIPKHTQLQDAHAGERPPKKLKLSQETTEQ----DIDPTNTEQRPL-EL
Sb_05g001150 275 -------------------------KPAASIAPPQPAVMDNIPKHTQLQDAHTGERPPKKLKLSQVTSDQ----DMDPETTEQRPL-EL
Os_IBM2 260 -------------------------VSKSTEN-----------ITKPAQKVLPGERPPKRVKFSENVTVQNVPLDVPERPSRTGPL-EL
AT3G15605 216 -----------------------PVNTETTTRGQIPILKTRAFGELYASGGSSVNAKPSKKRKLILNTPETDDSDDQGPQSGEKKLIKNP
RRM
Pt_0006s26040 437 HIQEVTRRP---------EAPWEERMQTAHEQGTLVLLQNLDPSYTSAEVEDIIWHAFKQSCTVKMIQRTASSPHSGQAFVIFQKREVA
Pt_0018s00870 354 KIQEPTRRP---------ATPWEETMQTAHEQGTLVLLQNLDPSYTSAEVEDLIWQAFKQSCTAKMIQRTARSSPHSGQAFVIFQKREVA
Rc_0803370 531 KILEVTRRPEADKIKWFKGLPWEERIKAAHAEGRIVLLQNLDPSFTGLEVEDIVWHALKQSCTAKMIPCTAFSSPHSGQAFAIFKTREAA
At_IBM2 453 QVTEVCRRPDAGKSKWFRSLPWEESMREAEKKGTVVLLQNLDPTYTSDEVEDIVYSALNQQCEARMIERTSVTIPHIGEALVIFKTREVA
Zm_GRMZM2G04486 338 QPKNIDRSK-------WFNIEWNDRLKMADDKGTLVYIQNLDIRFGPADIEELVRKALQLNCTAKPINHPTYDDPNNGKAYAIFRTKDAA
Sb_05g001150 334 PPKNIDRSK-------WFNIEWDDRLKMADDKGTLVYIQNLDIRFGPADIEELVRKALQLSCNAKPINHPTYDDPNNGKAYAIFKTKDAA
Os_IBM2 312 AGRQADRSK-------WFKIPWDTRLRNADEQGTLVYIQNLDIQFAAADIEELIRDALQLNCIAKPINHPTYDDPNNGKAYAIFKTKSAA
AT3G15605 282 PLVEKAPSQNIEKKSWYKKLPFEDELKPAIEKGRVVLLIENLEPSYTSLEVEFLFRQAFKEGVDAKMIPSSPMSSPHSGRALVIFGTKAA
Pt_0006s26040 518 EMAVAKLDEGCLMLSNGRYGFTLFQHCIAAPCFPGKQSTFFGHLVINKLRIHKQR--EMKEAVSTSHCSQPNTLEYEMAMDWCLLQERSD
Pt_0018s00870 435 EMAVAKLDEVCLMLSNGRP----LVGSIAAPCFPGKQSTFFGHLTINKLRIHMQR--EMKEAVSTSHCSQPNTLEYDMAMEWCLLQDRSD
Rc_0803370 621 ETAVTKLDEGCLMTSYGRP----LVGSIAKLSFPGKPSTYFGHMSIDKTRHQMQR--VMREAVSTSHCSQPNTIEYDMALEWCFLQEQTN
At_IBM2 543 ERVIRRLDEGCLLLSSGRP----LVASFAKITPPGKPSLFSGHIKLHKTQTR--R--EMRDAVATSHSSQPNNLEFDMAMEWCLHQARHE
Zm_GRMZM2G04486 421 DAAISKINSGLVVG--GRP----LYCSKGLLEVPKPSANLVGHLSS-YVKIGQKQRDEQKKAVATSHCSQPNTIEYDLALDWMLLREKQD
Sb_05g001150 417 DAAISKINSGLVVG--GRP----LYCSKGLLEVPKPPKNLVGHLSS-HVKIGHKQRDEQSKAVATSHCSQPNTIEYDLALDWMLLREKQD
Os_IBM2 395 DSAISKINSGLVVG--GRP----LYCSKGLLKVPKPSETLLGHLTINNIRMGIRQREEQKKAVSTSHCSQPNTMEYDLALDWMLVRAKQE
AT3G15605 372 DSAMSRLNEDCLMLSGQRA----LTGSKNVPVEIGRCRSFTGHFNMVDR--------SLMTAQKVSCCITLRLFSVVISLVMVTHRKPNS
gRNA2
Pt_0006s26040 606 LALRKLRQQQRQELRKLWVTLKCK--
Pt_0018s00870 519 LALRKLRQQQEQELRKLRATLKSK--
Rc_0803370 705 QEWELLYKQHGKELRKLESTLTRQAS--
At_IBM2 625 QASESVSKRQLEEMKSLRINFKLKLPYETQEFWFHAIQSPNFDSEEYTDAASGLKSDDPFVVTYFFVRVSFTSEVLNFCVDQERSRE
Zm_GRMZM2G04486 504 QSFRVLHKKHREARKGFASLVGKGSKAE--
Sb_05g001150 500 QSFRILHKKHREARKVFASLGNKSLKAEK---
Os_IBM2 479 TKFRTLHKKHKDERKTFASKMGK---
AT3G15605 450 PCQITEYYKLTPLPFSLIIVNRPHKFVNFQPHP---
