# Supplementary material for: Transcriptional regulation of genes bearing intronic heterochromatin in the rice genome
Source: PLoS Genet. 2020 Mar 18;16(3):e1008637. doi: 10.1371/journal.pgen.1008637 (PMC7145194; doi:10.1371/journal.pgen.1008637)
Supplement: S11 Fig — (A) Whole plant picture of three-month-old Nipponbare (left), RNAi_#2 line (middle) and RNAi_GFP control line (right). (B) Close-up pictures of seeds set in Nipponbare and RNAi lines (T1). (C) A close-up picture of seeds set in osibm2_g2#24 and their segregating wild-type siblings (WT; T4). White bar: 1 cm. (D) RT-PCR analysis of gene expression in endosperm and embryo of Nipponbare and osibm2. RNAs from ~10 DAF (Days After Fertilization) developing endosperm and embryo of osibm2_g2 #24 (T2) were used for the analysis. (PDF) [file pgen.1008637.s011.pdf]

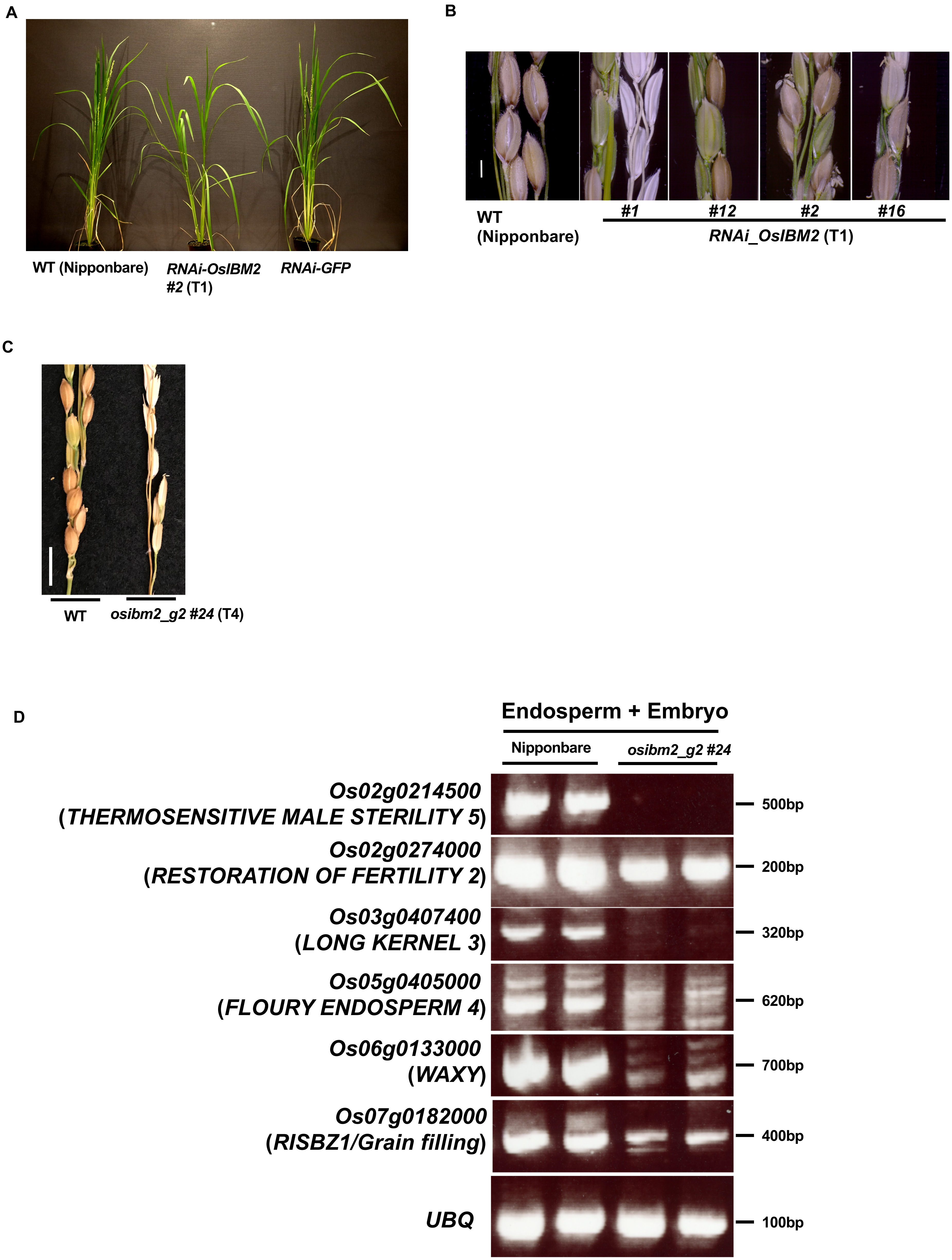
A
WT (Nipponbare)
RNAi-OsIBM2 #2 (T1)
RNAi-GFP
B
WT (Nipponbare)
#1
#12
#2
#16
RNAi_OsIBM2 (T1)
C
WT
osibm2_g2 #24 (T4)
D
Endosperm + Embryo
Nipponbare
osibm2_g2 #24
Os02g0214500 (THERMOSENSITIVE MALE STERILITY 5)
500bp
Os02g0274000 (RESTORATION OF FERTILITY 2)
200bp
Os03g0407400 (LONG KERNEL 3)
320bp
Os05g0405000 (FLOURY ENDOSPERM 4)
620bp
Os06g0133000 (WAXY)
700bp
Os07g0182000 (RISBZ1/Grain filling)
400bp
UBQ
100bp
