# Supplementary material for: Transcriptional regulation of genes bearing intronic heterochromatin in the rice genome
Source: PLoS Genet. 2020 Mar 18;16(3):e1008637. doi: 10.1371/journal.pgen.1008637 (PMC7145194; doi:10.1371/journal.pgen.1008637)
Supplement: Attachment — Submitted filename: Comments to the Authors191213.docx [file pgen.1008637.s025.docx]

**Responses to reviewer’s comments**

First of all, we would like to thank the reviewers for their constructive and valuable comments with regard to our manuscript. We have indicated the major changes made in the revised manuscript, and further provided point-by-point responses to the reviewers’ comments below.

**Additional results and major changes**

-As suggested by the reviewer #1, WGBS data have been re-analyzed using different parameters. A summary table of the new WGBS analysis was added as Supplementary Table S5. As the result of new analysis, the number of genes containing heterochromatic introns has been changed from 4150 to 4227. Accordingly, subsequent analyses have been performed again using the updated list of genes containing heterochromatic introns related to WGBS, and made changes in the relevant Figures; Fig.1 A-E, Fig.2 A-C, Fig.3 C-E, Fig.4 A-C, Fig.5 E, F, Fig.S1B, Fig.S4, Fig. S5, Fig. S6, Fig. S7, Fig. S13.

-In response to the comment by reviewer #2, positions of TE insertion in introns were analyzed and a new figure was added as Fig. S2C.

-In a response to the comment by reviewer #2, DNA methylation data of heterochromatic introns were added as Fig. S3C.

-As suggested by the reviewer #3, GO depletion analysis was added as Fig. S7B.

-We added new data for the analysis of stress responsiveness of genes in various environmental conditions using a public RNA-seq data, which were added to Fig. 4

-For the JA treatment analysis, we increased the number of genes that have structural variations in heterochromatic introns in KASALATH background as suggested by reviewers. We examined the responses of 12 genes with intronic variations to JA, of which only 4 (out of 12) showed a reduced response to JA treatment. Therefore, in the revised manuscript, we have de-emphasized the argument regarding a regulatory role of intronic heterochromatin in the rice genome, and also modified the title of the manuscript. The figure was moved from main to Supplementary Figures S8 and S9.

-In a response to the comment by reviewer #3, RT-PCR analysis of genes in *osibm2* endosperm and embryo was performed and the results were added as Fig. S11D.

-RNA-seq data were re-analyzed with a new pipe-line and a summary table was added as Table S6. This changed the number of differentially expressed genes in Fig. 5E.

-In a response to the comment by reviewer #2, we further analyzed the differences between differentially expressed genes (DEGs) with and without heterochromatic introns. We found that DEGs with heterochromatic intron showed a significant reduction of 3’ transcripts compared to DEGs without heterochromatin. The data were added as new Fig. S12.

- As suggested by the reviewer #3, expression analysis of TEs in *osibm2* mutant was performed, and the result was added as Fig. S14D.

**Point-by-point response to the referees’ comments**

*Reviewer #1: The authors characterise heterochromatic introns (defined as introns with 5 or more consecutive mCHG with average methylation >=0.5) in the rice genome, and demonstrate that the OsIBM2 gene plays a similar role to its Arabidopsis counterpart in the transcription through heterochromatic regions, but unlike in Arabidopsis OsIBM2 mutations have severe phenotypic consequences. The manuscript is clear and interesting. My comments are therefore mostly technical. The conclusions about regulatory roles of intronic heterochromatin can be de-emphasised, while the data from OsIBM2 knock-downs and knock-out is very nice and could have a greater place in the abstract.*

Major comments:

*1. The evidence for the regulatory roles of intronic heterochromatin is too thin to include it as a conclusion in the abstract. The sentence in the summary (“may have regulatory roles...”) is more accurate, and should be preferred.*

**Our response: As suggested, we have rephrased the texts in the title, abstract and results to de-emphasize the regulatory role of heterochromatic intron.**

*2. Part of the evidence comes from a GO analysis. GO enrichment results are in general difficult to interpret, but it is even more difficult here as the fold-enrichments and number of genes of each category are not reported. The broad term “catalytic activity” is significantly enriched, but how many of the 2,500 genes with heterochromatic introns are concerned?*

**Our response: In the new analysis of WGBS data, we found that 2449/4227 genes with heterochromatic introns have GO terms. The number of genes in each category and background were indicated in Fig. 4A, as suggested.**

*3. Additional evidence comes from the entropy of gene expression. Although there are significant differences between genes with and without heterochromatic introns, the magnitudes of the difference seem very small. The text should reflect this.*

**Our response: We have reanalyzed the entropy of gene expression in various environmental conditions using a public RNA-seq data, which has a more comprehensive data set. The results were consistent with the previous ones, where genes with heterochromatic introns tend to respond to hormone treatments such as JA and ABA. In addition, we added new results showing that the genes with heterochromatic introns are also responsive to abiotic stresses such as cold and flood (Fig. 4C and S7B). However, only treatments showing significant changes with p<1e-5 were retained in the Figures, as suggested.**

4. The evidence around the JA responsiveness is very weak. From just 4 cultivars and 3 genes with no consistent response patterns across cultivars, I believe the carefully-worded conclusion that “these results suggest that structural variations of heretochromatic introns may have impacts on gene responses to environmental signals” is still too strong: it may also have no impact. One would need a lot more cultivars to start deriving trends, and/or a lot more JA-responsive genes. I recommend keeping the results in the manuscripts but noting that they do not provide evidence. The authors rightly note that non-intronic sequence polymorphims may also explain the altered JA response; trans-factors may also be at play.

In presenting this data, plotting individual points rather than mean + sd would be preferable. The use of asterisks is better kept for statistical significance than category of structural variation. Please also give the results of the multiple-testing-corrected statistical tests.

**Our response: We increased the number of genes that have structural variations in heterochromatic introns in the KASALATH genome background, and eventually examined JA responses of 12 genes with intronic variations. Although several (4 out of 12) genes with intronic variations showed a reduced response to JA treatment (p>0.05), we could not observe significant changes in other loci in response to the treatment. Therefore, in the revised manuscript, we de-emphasized the argument regarding a regulatory role of intronic heterochromatin in the rice genome, and also reworded the title of manuscript. As suggested, dot plots were used instead of bar graphs for presenting gene expression levels, and the figure was moved from the main to Supplementary Figs. S8 and S9.**

Minor comments:

*1. Please provide a summary table for the sequencing (WGBS and RNA-seq): number of reads sequenced and mapped, bisulfite conversion rate, median cytosine coverage, etc. It will help in quickly assessing study design and robustness of the underlying data.*

**Our response: As suggested, summary tables for WGBS and RNA-seq were added as Supplementary Tables S5 and S6, respectively.**

*2. The mapping strategy for the bisulfite data may cause a small amount of double-counting: if the reads of a pair overlap each other, and map to a SNP (or PCR error), they would not be aligned in paired end (no mismatches allowed) but would both be successfully aligned in the second round as single-end reads with 1 mismatch. The cytosines in the overlapping region would then be counted twice. This should be rare and is unlikely to affect the results, but I’d recommend relaxing the mapping parameters in the paired-end round. Here again the summary table of mapping efficiency/... would help in evaluating the mapping strategy.*

**Our response: As suggested, we reanalyzed our WGBS data with the suggested parameter settings, i.e. allowing 1 mismatch in PE and SE alignments. We also used more sensitive bowtie2 settings as described in Methods. After excluding methylated Cytosnes (mCs) outliers as suggested in the comment below, eventually ~3% more mCs were called than the previous analysis. A summary table was provided as Supplementary Table S5. This resulted in the detection of more heterochromatic intron genes (from 4150 to 4227 genes). Related figures were replaced with the new gene data accordingly.**

*3. Cytosines with low coverage (0-2) are excluded, but it may also be worth to exclude outliers at the upper end: regions of unnaturally high coverage may be chloroplast insertions in the genome assembly or, particularly relevant to this study, repeated elements that are not well represented in the assembly*.

**Our response: As suggested, we excluded cytosines that covered by more than 100 reads (top ~0.01%). Details are provided in Supplementary Table S5.**

*4. It is unusual to deduplicate RNA-seq reads, in the absence of UMIs it risks introducing biases (for a discussion see https://sequencing.qcfail.com/articles/libraries-can-contain-technical-duplication/ and https://www.biostars.org/p/55648/). Did the libraries require this? If they did, any results obtained after deduplication should be taken with a pinch of salt.*

**Our response: We rechecked our RNA-seq pipeline, and found that the deduplication of RNA-seq reads was applied only for RPM calculation to visualize read tracks such as Fig. 1E, while for the differential expression analysis, we did not deduplicated reads. This was stated in the Methods section.**

*5. It is also unusual to collapse biological RNA-seq replicates (l. 524-528). Please keep the replication in the analysis even if it means relaxing the significance threshold to look at an OK number of genes.*

**Our response: As suggested, we applied a statistical method to analyze data from biological replicates, and detailed the changes in the Methods section. We also relaxed the threshold for detection of genes showing changes in the mutants from q<0.001 to 0.01, which resulted in 454 genes detected as DEGs. (Fig. 5E).**

*6. TPM usually refers to Transcripts Per Million rather than Tags Per Million. If the authors mean tags per million, the cpm (counts per million) unit would be clearer.*

**Our response: We meant TPM as Transcripts Per Million, as mentioned by the reviewer. We changed the wording throughout the manuscript.**

*7. Plots in Fig 4B and C are the showing the same thing, only with a different axis-scale. It’s a good idea to plot log values of cpms/tpms rather than non-logged counts, as in Fig4C. If the style of Fig4B is desired, a violin plot would advantageously summarise all the information into one graph.*

**Our response: As suggested, we changed Fig. 4B to a boxplot with log10(TPM), and the original Fig. 4C has been removed.**

*8. In the context of their study, the authors discuss the documented link between R genes and TEs. Could this be specifically addressed? For instance, are R genes over-represented in the set of genes with intronic-heterochromatin?*

**Our response: We found that ~2.8% of genes with heterochromatic introns are R-genes, which are significantly overrepresented compared with the background (*p* < 0.001, Fisher’s exact test). This was added in the Discussion section.**

Reviewer #2: This manuscript provides a comprehensive analysis of introns with heterochromatin in rice. Given that we know very little about this in larger genomes, and that the authors did comparative analysis with other rice cultivars, and that they included mutant analysis, I was initially excited about these results. The idea that intronic TE insertions may be an important source regulator information is indeed intriguing, but I do not think the authors have made the case, nor have to considered numerous alternative interpretations of their data. The mutant analysis was particularly disappointing, as only a tiny proportion of any of the genes’ expression was affected in the expected way and a more comprehensive analysis of the RNAseq of the mutants was missing. In the end, the authors ended up with anecdotes that may or may not support what is clearly a favored hypothesis. However, particularly when it comes to TEs and epigenetics, we must always consider the null hypothesis, which is that most TEs most of the time do not provide a selective advantage to their host. The authors are have made some intriguing arguments based on their data, but, in the end, it is not convincing.

Line 68: “often acquire regulatory functions for surrounding genes”. Unclear what this means.

**Our response: We changed the sentence to “often affect transcription of surrounding genes”.**

Line 113: “introns at genome-wide” should read “introns at a genome-wide”

**Our response: We changed the sentence as suggested.**

Line 159: or preferential targeting? Many TEs appear to preferentially target the 5’ ends of genes.

**Our response: We further analyzed the position of introns containing TEs and a figure was added an extra figure as Fig. S2C. As commented by the reviewer, TE insertions were highly enriched in the 1^st^ and 2^nd^ introns, associated with the heterochromatic state. This was reflected in the main text as “, which is associated with accumulation of TEs in promoter proximal introns (Fig. S2C). This suggests that a preferential targeting of TEs toward the 5′-ends of rice genes might be a trigger for the formation of heterochromatin in promoter proximal introns.”**

*Line 165: Hopefully, this data set will be made available.*

**Our response: We added a sentence, “The list of TEs is available upon request.” in the Methods section.**

Line 178: This is very likely the case. Are these repetitive or single copy? Do they form hairpins? Are they helitrons, which are notoriously difficult to identify?

**Our response: We further analyzed DNA methylation of heterochromatic introns with and without TEs/repeats (added as new Fig. S3C). The result showed that DNA methylation at heterochromatic introns with TEs/repeats is relatively confined to the intronic regions, while DNA methylation is high in the surrounding regions of heterochromatic introns without TEs/repeats. This suggests that DNA methylation of the heterochromatic introns is affected by the surrounding epigenetic states. Indeed, we found that 65% (419/639) of genes containing heterochromatic introns without TEs/repeats are overlapping with repeat annotations, which may suggest miss-annotation of TEs as genes.**

**The repeat annotation that we used (downloaded from RAP-DB (**[**https://rapdb.dna.affrc.go.jp/download/irgsp1.html**](https://rapdb.dna.affrc.go.jp/download/irgsp1.html)**) contains 761 annotations of RC/Helitron, and only 1 intronic region overlapped with the annotations.**

**For hairpin structure prediction within the heterochromatic introns, we used the software DNA PUNCTUATION (http://www.dnapunctuation.org) with the following parameter; stem length 15-50 bp, loop 3-10 bp, with 3 mismatches/gaps allowed. We randomly chose 100 heterochromatic introns (total ~60kb in length), and found ~40 hairpin structures in the heterochromatic introns without repeat/TE annotations (~0.67 loci/kb). However, we found a similar frequency of hairpin structures within non-heterochromatic introns (~0.5 loci/kb) with the identical parameters. Since these hairpins did not associate with heterochromatic features, we could not conclude that hairpin structure alone is sufficient for triggering heterochromatin formation, and these results were not added in the revised version of the manuscript.**

Line 246: I’m not sure I find these data particularly convincing. It is a small number of genes and, as the authors state, there certainly could be other causal sequence polymorphisms. It would have been more convincing with more examples, perhaps of other enriched terms? Also, the error bars from the RT-PCR are pretty large.

**Our response: We increased the number of genes that have structural variations in heterochromatic introns in the KASALATH genome background. We eventually examined the JA responses of 12 genes with intronic variations. Although some (4 out of 12) genes with intronic variations showed a reduced response to JA treatment, we could not observe a consistent response of other loci to the treatment. Therefore, in the revised manuscript, we have de-emphasized the argument regarding a regulatory role of intronic heterochromatin in the rice genome, and changed the title of the manuscript. We also used dot plots and error bars instead of bar graphs for presenting gene expression levels, and the figure has been moved from the main manuscriptto Supplementary Figs. S8 and S9.**

Line 279: I’m having a hard time interpreting this. “detected 198 genes both with and without heterochromatic introns that showed changes in transcripts downstream of introns (27%; 54/198 genes with heterochromatic intron, expression of which commonly changed in RNAi_#2, #16 and osibm2_g#24 lines; Table S3)”. So a total of 198 showed changes and some were with and some were without heterochromatic introns. And only 27% of the total were heterochromatic. Is this statistically significant? Since 11% of introns are heterochromatic, I guess it could be, but the argument should be made here. And the percent of heterochromatic introns that are affected was only 54/4,150, or 1.3%. I may be missing something, but this result does not appear to represent a trend.

**Our response: We reanalyzed our RNAseq data according to the suggestions of the reviewer #1. Our RNAseq data were obtained using the leaf tissues, and expression of 2640 genes out of 4227 genes containing heterochromatic introns were detected (Fig. 4B). We also relaxed the threshold for the detection of genes showing changes in the mutants from q<0.001 to 0.01, which resulted in the detection of 454 genes as DEGs, and 93 out of the 454 genes (20.5%) were found to contain heterochromatic introns. Although this proportion was less than the previous analysis (27%), genes containing heterochromatic intron were still significantly enriched among DEGs (93 genes out of 454 DEGs (20.5%); *p*=2.9e-17, Fisher test).**

**There are several explanations for the small number of genes detected in our RNAseq analysis. First, we used RNAi lines and the CRISPR lines that were most likely partial loss of function mutants, as we could not obtain homozygous deletion lines for the BAH domain in the N-terminal part of OsIBM2 gene. Therefore, we could only analyze the *osibm2* mutants with less severe effects on target genes. Second, *osibm2* mutants showed relatively mild morphological phenotypes in the leaf tissue compared with the endosperm and embryo (Fig. S11), and tissue-specific genes or stress responsive genes in certain conditions could not be detected by our RNAseq dataset using the leaf tissue. Indeed, additional RT-PCR analysis using these tissues detected expression changes of genes containing heterochromatic introns (Fig. S11D), even though they were not detected as DEGs in the RNAseq dataset of leaf tissue. These arguments have been added to the main text of the revised manuscript.**

Line 283: But the same could be for the non-heterochromatin intron genes.

**Our response: As commented, the sentence was revised as “The number of DEGs with and without heterochromatin ~’’.**

Line 288: Why not do the control, which are genes whose downstream expression went down even though they didn’t have any heterochromatin?

**Our response: As suggested, we compared the degree of changes in the 3’ region of DEGs with and without heterochromatic introns (Fig S12B). We found that the genes with heterochromatic intron showed a significant reduction in transcription. DEGs with normal introns showed less changes as a group, even though they were detected as DEGs individually. This suggests that the *osibm2* mutation dominantly affected the DEGs with heterochromatin.**

Line 289: I disagree, what the data suggests is that a very small fraction of genes with and without heterochromatin are affected when OsIBM2 is knocked down.

**Our response: The sentence has been removed from the revised manuscript.**

Line 295: Which allele was this?

**Our response: The data were obtained using the CRISPR mutant *osibm2*_*g#24.* This has been added to the sentence.**

Line 326: In plants with large genomes, many gene models do not actually correspond to genes. Rather, they represent Pack-MULEs and helitrons, that capture gene fragments. If they were inadvertently counted, then this would explain an overall relaxation in selection. Alternatively, genes under relaxed selective contraints may simply be able to tolerate these insertions.

**Our response: We appreciate the reviewer’s comment. We found four of those transposases in genes without heterochromatic introns (i.e., in controls). We reanalyzed the data by excluding these genes, and obtained essentially the same results.**

Line 332: Don't you mean exon here? You can’t get Ka/Ks from introns.

**Our response: Ka/Ks was calculated by amino acid substitution of genes with/without heterochromatic introns.**

Line 343: I’m not convinced of this. The mutants could cause lethality for a variety reasons, and only a minority of genes that were changed downstream of the introns had heterochronic introns, as the authors point out. Further, I’m guessing that expression of a very large number of genes besides these were affected in the mutant.

**Our response: As commented, we still do not know the major cause of the sterility observed in the *osibm2* mutants, even though additional RT-PCR showed that some genes essential for reproductive development were affected in *osibm2* (Fig S11D). Nonetheless, transcriptions of dozens of heterochromatic genes were affected in *osibm2* with the unique phenotype, i.e. premature termination of transcripts at heterochromatic introns (Figs. 5, S11, 12, 13, 14). We rephrased the sentence as “transcriptional regulation of genes by OsIBM2 is essential for rice development and reproduction. For plant genomes harboring abundant intragenic heterochromatin, gene regulation mechanisms involving IBM2 would be more vital”.**

Line 359: Alternatively, some genes, such as those expressed at lower levels or under certain conditions may simply tolerate these insertions.

**Our response: We have incorporated the comment in the paragraph.**

Line 376: There is also ample evidence that many Ts target the 5’ ends of genes, and insertions into the exons in the 5’ ends of genes would be selected against.

**Our response: We have incorporated the comment in the paragraph.**

Line 389: I don’t think so. It just shows that neutral sequences evolve rapidly, and methylation makes them evolve more rapidly.

**Our response: As commented, we modified the sentences and emphasized the effect of DNA methylation on nucleotide substitutions.**

Reviewer #3: The manuscript by Espinas and colleagues reports on the description of heterochromatic introns (HIs) in the rice genome. The authors have performed WGBS to reveal introns with heterochromatic signature (DNA methylation in all sequence contexts) and found around 4000 genes harboring such HIs. They further analyzed the epigenetic control of these introns, and obtained mutants (RNAI lines and CRISPR mutant) for IBM2. The mutants are sterile suggesting embryonic lethality. However only 200 common genes are affected in the 3 lines analyzed. While the study is of interest I think the manuscript would benefit from a more complete description of some results.

*1. From Figure 3C it seems that repeats are enriched (compared to TEs) at HIs. Could the authors precise how were these repeats annotated? Concerning the authors' own annotation of TEs, how does it compare with published ones? (What % of the genome is covered?).*

**Our response: The repeat annotation was downloaded from RAP-DB (**[**https://rapdb.dna.affrc.go.jp/download/irgsp1.html**](https://rapdb.dna.affrc.go.jp/download/irgsp1.html)**), along with other genome annotations. In this annotation, repeat sequences were detected using the Censor software (**[**https://www.girinst.org/censor/**](https://www.girinst.org/censor/)**) using known repeat sequences in the rice genome. This repeats annotation covers about 42.7% of genome sequence (159.5MB/373.2MB). Our TE annotation was manually curated as described in the Methods section, which covers 15.1% of the genome, 94.1% of which overlaps with the repeat annotation.**

*2. Figure 4: what are the functional categories depleted for HIs?*

**Our response: As suggested, we further analyzed GO terms which are depleted among genes with heterochromatic intron. We found that several terms related to nitrogen metabolisms are depleted in the genes, and this is now mentioned in the main text. The full list of terms with the actual number of genes were added as a new Fig. S7B.**

3. Study of polymorphisms at HIs: why did the authors limit their analysis to 3 varieties when 3,000 rice genomes are available? Did they compare with the 12 rice species with assembled genomes?

**Our response: In this analysis, we aimed to find genes with structural variations within introns to examine the differential responses to JA treatments, and a large scale analysis of intronic variation was not in the scope. In the original manuscript, we manually searched those genes using a public genome resequencing data from a limited number of strains that overlapped with our rice seed stocks (Fawcett et. al., 2014). In the revised analysis, we exploited the INDAL data provided by the 3000 rice genome project (**[**https://snp-seek.irri.org**](https://snp-seek.irri.org)**) between Nipponbare and KASALATH as suggested, to comprehensively search structural variations in heterochromatic introns. We focused on the indica strain KASALATH, partly because other japonica strains are genetically too close to Nipponbare, and not many indels were found especially in the introns. Eventually, we listed about 20 loci and 12 of them confirmed by PCR were examined (Fig. S8, S9). However, the INDAL data-set from the genome project seems not to be comprehensive yet, as the large 2.5kb deletion in *Os11g0229300* was not listed in the dataset.**

4. The functional evidence based on only one gene (line 245) should be interpreted with great caution. Only CRISPR excision of the HI or introgression line could properly address this question. This validation would be beyond the scope of this study.

**Our response: We further increased the number of genes that have structural variations in heterochromatic introns in the KASALATH as mentioned above. We eventually examined the JA responses of 12 genes with intronic variations. Although some (4 out of 12) genes with intronic variations showed a reduced response to JA treatment (p>0.05), we could not observe a consistent response of the other loci to the treatment. We agree with the reviewer that deletions of these regions by genome editing would provide strong causal evidence towards the regulatory role of heterochromatic intron. In the revised manuscript, we have de-emphasized the argument regarding a regulatory role of intronic heterochromatin in the rice genome, and changed the title of the manuscript. The figure was moved from the main to Supplementary Figures S8 and S9.**

5. *IBM2 mutant analysis is not described in the abstract, not in the introduction. It should be mentioned as it represents an important part of the manuscript. Did the authors investigate the putative target genes involved in the sterile phenotype? What about TE expression in this mutant? I think this part of the work will be of interest to many rice researchers and could be described in more details. For instance how to explain the strong phenotype given that osibm2 has no impact on DNA methylation? Could the difference in overexpressed genes in the 3 lines be stochastic and depend on the level of IBM2 remaining expression (line 16 clearly the most affected)?*

**Our response: As suggested, we have added sentences about OsIBM2 analysis to the Abstract and Introduction.**

**Although the detailed analysis of the mechanisms by which *osibm2* causes sterility in rice would be the subject of future studies, we further examined the expression of several genes containing heterochromatic introns in the mutant endosperm/embryo, which are known to be involved in the reproduction and seed development in rice (added as new Fig. S11D). Although these genes were not detected as differentially expressed genes in our RNAseq analysis of leaf tissue, their expression was severely affected in the mutant seeds. Although we cannot conclude that the changes were due to a direct effect of *osibm2*, the observed sterility in *osibm2* mutants is likely due to expression changes of genes in multiple loci that are important for proper reproductive development.**

**In terms of TE expression changes in *osibm2* mutants, we searched differentially expressed TEs in the CRISPR mutant *osibm2* by DEseq2. We found only a small number of TEs (23 TEs; LTR 22, DNA/En-Spm 1) that showed significant changes (q<0.05) in both upregulation (12 TEs) and downregulation (11 TEs). Since the mutants were generated through tissue culture transformation, some of the expression changes could be due to epigenetic changes during tissue culture. We also detected 7 up-regulated intronic TEs, which are due to changes in transcription/splicing of intronic region in the *osibm2* mutants. The result was added as Fig. S14D.**

**The absence of changes in DNA methylation in *osibm2* mutants suggested that OsIBM2 acts downstream of intragenic heterochromatin, consistent with our previous observations in *ibm2* mutant in *Arabidopsis* (Le et al., 2015).**

**We could not conclude that the variability of gene expression changes among RNAi and CRISPR lines was due to the level of *OsIBM2* expression. As mentioned in the main text, these mutant lines including the CRISPR line were likely a partial loss of function mutants, while effects of RNAi are known to be variable in individual plants, and/or in different tissues. Nonetheless, the CRISPR mutant line (*osibm2_g#24*) showed consistent effects on gene transcription, compared to the RNAi lines (for example, Fig. 5G, Fig. S13).**

Minor comments:

Line 105 reference to be edited.

**Our response: We corrected the reference to adhere to the style of the journal.**

Line 106: please specify that this epiallele (Karma) is revealed under in vitro culture conditions

**Our response: As suggested, we added an explanation for the condition that caused the epiallele in the text.**

0s11g0229300 could be shown in Figure 1.

**Our response: Because of aforementioned reasons, we kept them in Fig. S8 and S9.**
