# Supplementary material for: Transcriptional regulation of genes bearing intronic heterochromatin in the rice genome
Source: PLoS Genet. 2020 Mar 18;16(3):e1008637. doi: 10.1371/journal.pgen.1008637 (PMC7145194; doi:10.1371/journal.pgen.1008637)
Supplement: Attachment — Submitted filename: Responses to reviewer.docx [file pgen.1008637.s026.docx]

**Responses to reviewer’s comments**

We thank reviewers for their time to review again our manuscripts and providing useful comments for improvement of the manuscript. We provided our responses to the comments below.

**Additional results and changes**

-As suggested by reviewer #1 and #2, the TE annotation used in this study was added as supplementary table S7.

-In the response to the reviewer #2, detailed methods for entropy calculation was added to the method section.

-In the response to the reviewer #2, a figure for loci containing differentially expressed TEs was added as FigS14E.

**Point-by-point response to the referees’ comments**

*Reviewer #1: The authors have made a substantial effort to address the comments. The data are better presented and the toned-down conclusions are more accurate.
I would still like to see a mention of effect size, and not only statistical significance, in the description of the GO enrichment and entropy analyses. For example, an 18% enrichment in cell death terms seems quite small. Overall it seems that the GO terms of heretochromatic intron-containing genes are not too different from the rest of the genes.*

*For the entropy (and overall expression), the changes also appear to be modest. For the R gene enrichment, please specify the “background” R gene percentage.*

**Our response: As suggested, effect sizes of data in the GO analysis (odds ratio) and the entropy analysis (r) were calculated and now indicated in the figure legends (Fig 4B, C, S7). Overall, the effect sizes of GO analysis were small to medium. In the entropy analyses, effect sizes with and without heterochromatin were small (r <0.1) despite the significance of p-values, which is likely due to the relatively large number of sample sizes used in the analyses. We mentioned the effect size of the analysis the main text, and further deemphasize the arguments regarding specific biological pathways and GO terms in both the abstract and throughout the main text of the revised manuscript. We also added the background numbers of R gene in the discussion.**

*As requested by reviewer 2, the TE annotation should be deposited rather than relying on direct requests.*

**Our response: As suggested, we added the TE annotation used in this study was added as supplementary table S7.**

*There is a small formatting error on the p-value on l.161.*
**Our response: We corrected the error.**

Reviewer #2:

*Overall, the authors have done a good job addressing the concerns I had. However, I do have some remaining issues that should be addressed before publication.

General note: Since the locations of the TEs is essential for replicating these experiments, I do not think that making the TEs “available on request” is sufficient. The data should be provided as a supplemental data set in an easily convertible format (not a PDF). Also, please be sure to address my confusion concerning entropy values for the various conditions.*

**Our response: As suggested, we added the TE annotation used in this study as supplementary table S7.**


Line 39. Not sure what “basal functions” means here.
**Our response: We removed the words from the revised manuscript.**

Line 53. And yet most genes with h-introns are not affected by the mutation.

**Our response: We changed the sentence to “a group of heterochromatin-containing genes”.**

*Line 66. “ species with small genome” should be small genomes*

**Our response: We corrected the word.**

*Line 95. You should say what IBM2 is here.*

**Our response: As suggested, we added a description of IBM2 in the text.**

*Line 144. should read, “similar to”*

**Our response: As suggested, we corrected the word.**

*Line 210. Figs. should be Fig.*

**Our response: As suggested, we corrected the word.**

*Line 223. I find this a bit confusing. How is the entropy for the treatments work? Were multiple tissues for each treatment examined? If not, how was entropy calculated? By just comparing treated and untreated? If you are talking about “responsiveness” wouldn’t you want to look at changes in expression (up or down?).*

**Our response: We regret that we did not provide detailed descriptions for the entropy analyses in the previous versions of the manuscript, and thank the reviewer for raising this point. For gene expression of developmental stages, micro-array gene expression profile of 48 rice developmental stages/tissues was retrieved from RiceXpro database [63], and entropy values (modified H) for each gene using the median value of replicates were calculated by TCC package in R [120]. Generally genes showing tissue/condition specific expression patterns (either upregulated or down regulated) have lower entropy, while ubiquitously expressed genes (such as house-keeping genes) have higher entropy. For expression profiles of stress/hormone treatment conditions, RNA-seq data was retrieved from** **TENOR database [54]. The expression data from different time points for each experimental condition were used: Jasmonic Acid, ABA, Cold, Drought treatments; 0, 1, 3, 6, 12, 24 hours, Flood; 0, 1, 3, 6, 12, 24, 72 hours, Osmotic stress; 0, 1, 3, 6, 12 hours, High/Low phosphate treatments; 0, 1, 5, 10 days. These descriptions were added in the method section of the revised manuscript. As mentioned in the responses to reviewer#1, effect sizes of the data were small, we deemphasized the argument about responses to those stimuli in the revised manuscript.**

*Line 229. looked for, or sought, rather than sought for.*

**Our response: As suggested, we corrected the word.**

*Line 275. So expression of the downstream exons relative to upstream exons, or relative to w.t.?*
**Our response: They were compared with the 5′/3′ ratio of wild type transcripts. This was added in the text.**

*Line 276. Given that the degree of knockdown for the two RNAi lines are nearly identical, why is there a massive difference between the two with respect to differential expression of downstream exons? Also, just to be clear, these are all DEG with respect to the ratio of 5’ to 3’ exons, not all the DEGs?*

**Our response: The reason for the different effects of the RNAi transgenic lines was not clear, although the efficiency of knock-down would be variable in individual plants, and/or in different tissues. Also, RNAi _*#16* had only one replicate, which resulted in the detection of a larger number of DEGs. For the 5′/3′ ratio calculation, DEGs mapped with sufficient amount of RNA-seq reads (TPM>1) were used.**

*Line 281. Since I can’t tell where the primers are and the “truncated” transcript looks like a smear, I am not convinced that figure 5G shows premature polyadenylation. Figure 5F is confusing, since it shows that 610300 (OsIBM) has a loss of 5’ exons in plants targeting the 3’ exons. However, the overall trend, for downstream exons to be lost (presumably through premature polyadenylation) is clear and convincing, and the examples in Figure S14 are more convincing (perhaps one of these should be used in place of the one in the main figure).*

**Our response: For the 3’ RACE experiments, 5’ gene specific primer positions were indicated as arrowheads in the upper panel of the figure. The 3’ primer was an universal oligo-dT primer and not specific to any loci. For Fig. 5G, we replaced the gel picture of *Os01g0650200* with a picture with clearer band image. In addition, we exchanged the Fig. 5G with Os03G033210 in Fig.S14A, as suggested. Transcript peaks in the 3’ part of OsIBM2 loci were likely from the RNA of hairpin transgene (position was indicated in Fig.5A and the bottom track of Fig.5F) used for RNAi, while the decreased 5’ transcripts reflected the levels endogenous OsIBM2 transcripts.**

*Line 287. I’m assuming that none of these were associated with premature polyadenylation?*

**Our response: We found that 3 out of the 8 intronic TEs were forming heterochromatin and associated with the detected DEGs with a reduction of 3’ transcripts, suggesting that transcripts changes of these TEs were due to premature polyadenylation in the mutants. Track figures of these genes containing the TEs were added as Fig. S14E.**

*Line 316. Indeed, the TE insertion is even missing from the A. lyrate homolog of IBM1, so the global effects of IBM2 are almost certainly contingent on a random insertion into IBM1 in A. thaliana.*

**Our response: The heterochromatic domain in the intron of the *Arabidopsis* *IBM1* is associated with an insertion of organelle-like DNA sequence, which seems to be conserved in the *A. lyrata IBM1* (Saze et al., 2013, Fig. S12). However, whether the intron in *A. lyrate IBM1* is associated with heterochromatin is not clear yet. We added words to mention this in the beginning of the paragraph.**

*Line 333. should read constraint. Interestingly, the same appears to be true for upstream insertions of TEs in maize.*

**Our response: We corrected the word as suggested.**

*Line 341. What if you masked the methylated regions?*

**Our response: Since the nucleotide substitution rate was calculated by excluding repeat sequences in the introns, we assume that the value reflects the substitution ratio mainly at unmethylated sequences. However, there may be a certain effect of methylation spreading to surrounding sequences.**

*Line 352. This is hardly surprising given that many genes are presumably indirectly affected by the mutant.*

**Our response: We did not particularly mean to emphasize the part in the context.**

*Line 363. Not to quibble, but I often find this kind of argument frustrating. The implication is that heterochromatin formed at TEs is “functional” because premature polyadenylation at the TE in the IBM2 mutant occurs. However, I would argue that the IBM2 protein simply masks the presence of the TE so that it can be tolerated. What would convince me otherwise would evidence that the TE insertion in wild type plants has an effect on any aspect of the phenotype.*

**Our response: Although we did not mean to argue about “functional” TEs in this paragraph, we rephrased the sentence to “~transcriptional regulation of genes by OsIBM2 affects rice development and reproduction”.**

*Line 383. I never bought that argument, unless you think that maize has a far more sophisticated regulatory apparatus than does arabidopsis.*

**Our response: We removed the sentence in the revised manuscript.**

*Line 420. Selective pressure against?*

**Our response: We added the word as suggested.**

*Line 425. Yes, but aren’t these wild rices at least partly outcrossing?*

**Our response: Indeed, the wild rice strains they analyzed are outcrossing, although the outcrossing rates range from 5 to 50%. Interestingly, the report [103] showed that the genome sizes of the cultivars are larger and contain longer TEs than the wild rice genomes, while TEs were depleted in genic regions in the cultivar genomes.**

*Line 520. How could you get entropy values for this if you only looked at one tissue?*

**Our response: As described in the previous response, we added a detailed description of the analysis in the method.**
